# Utilizing the codon adaptation index to evaluate the susceptibility to HIV-1 and SARS-CoV-2 related coronaviruses in possible target cells in humans

**DOI:** 10.3389/fcimb.2022.1085397

**Published:** 2023-01-25

**Authors:** Haoyu Zhou, Ruohan Ren, Stephen Shing-Toung Yau

**Affiliations:** ^1^ Yanqi Lake Beijing Institute of Mathematical Sciences and Applications (BIMSA), Beijing, China; ^2^ School of Life Sciences, Tsinghua University, Beijing, China; ^3^ Zhili College, Tsinghua University, Beijing, China; ^4^ Department of Mathematical Sciences, Tsinghua University, Beijing, China

**Keywords:** codon adaptation index (CAI), HIV-1, SARS-CoV-2, translational efficiency, target cells

## Abstract

Comprehensive identification of possible target cells for viruses is crucial for understanding the pathological mechanism of virosis. The susceptibility of cells to viruses depends on many factors. Besides the existence of receptors at the cell surface, effective expression of viral genes is also pivotal for viral infection. The regulation of viral gene expression is a multilevel process including transcription, translational initiation and translational elongation. At the translational elongation level, the translational efficiency of viral mRNAs mainly depends on the match between their codon composition and cellular translational machinery (usually referred to as codon adaptation). Thus, codon adaptation for viral ORFs in different cell types may be related to their susceptibility to viruses. In this study, we selected the codon adaptation index (CAI) which is a common codon adaptation-based indicator for assessing the translational efficiency at the translational elongation level to evaluate the susceptibility to two-pandemic viruses (HIV-1 and SARS-CoV-2) of different human cell types. Compared with previous studies that evaluated the infectivity of viruses based on codon adaptation, the main advantage of our study is that our analysis is refined to the cell-type level. At first, we verified the positive correlation between CAI and translational efficiency and strengthened the rationality of our research method. Then we calculated CAI for ORFs of two viruses in various human cell types. We found that compared to high-expression endogenous genes, the CAIs of viral ORFs are relatively low. This phenomenon implied that two kinds of viruses have not been well adapted to translational regulatory machinery in human cells. Also, we indicated that presumptive susceptibility to viruses according to CAI is usually consistent with the results of experimental research. However, there are still some exceptions. Finally, we found that two viruses have different effects on cellular translational mechanisms. HIV-1 decouples CAI and translational efficiency of endogenous genes in host cells and SARS-CoV-2 exhibits increased CAI for its ORFs in infected cells. Our results implied that at least in cases of HIV-1 and SARS-CoV-2, CAI can be regarded as an auxiliary index to assess cells’ susceptibility to viruses but cannot be used as the only evidence to identify viral target cells.

## 1 Introduction

Pandemics of several kinds of RNA viruses including human immunodeficiency virus-1 (HIV-1) (Maartens et al., 2014) and severe acute respiratory syndrome coronavirus-2 (SARS-CoV-2) (Uddin et al., 2020) are one of the most important public health issues of our time. Diseases including acquired immunodeficiency syndrome (AIDS) and coronavirus disease 2019 (COVID-19) caused by these viruses have become major threats to the health of people all around the world. Because of the high transmission capacity, the huge number of infected patients and the lack of effective vaccines, attempts to control pandemics of these viruses are pretty difficult. Research on the pathological mechanism of related diseases and the development of effective antiviral drugs will become important strategies to fight against these viruses.

To understand the pathological process of diseases caused by viruses, the identification of target cells that are susceptible to them in humans is crucial. It is currently known that both HIV-1 and SARS-CoV-2 can infect different types of target cells in humans. For HIV-1, CD4+ T cells which aid the activity of other immune cells in the adaptive immune response are believed to be its major targets (Maartens et al., 2014). The decrease of CD4+ T cells is considered to be the major cause of immune deficiency in AIDS patients and a major prognostic marker for HIV-1 infection (Mellors et al., 1997). However, other types of cells have been reported to be susceptible to HIV-1. As an example, immune cells which belong to the monocyte-macrophage system (This term refers to a large group of cells that have the same origin during development and exhibit strong phagocytosis and antigen presentation ability during the immune response and other biological processes (Guilliams et al., 2014). Typical examples of this cell group include macrophage and dendritic cells in various tissues (Guilliams et al., 2014), Langerhans cells in the skin (Guilliams et al., 2014), osteoclasts in the bone (Kubatzky et al., 2018) and microglias in the central nervous system (Kim and Cho, 2016)) have been identified as target cells for HIV-1 (Coleman and Wu, 2009). Except for immune cells, other types of cells can also be infected by HIV-1. For instance, gastrointestinal symptoms like diarrhea and gastric hypoacidity are common in AIDS patients (Liu et al., 2013; Maartens et al., 2014). Besides the indirect effect of immune system dysfunctions, direct HIV-1 infection to gastric epithelial cells is also a possible reason for these symptoms (Liu et al., 2013).

For SARS-CoV-2, the problem of its target cells is more complex. Although COVID-19 is originally thought to be a respiratory infectious disease characterized by pneumonia, its symptoms can appear in various organs of the human body (Gupta et al., 2020). In the lung, type II alveolus epithelial (AT2) cells which are responsible for maintaining alveolus homeostasis are major target cells of SARS-CoV-2 in humans (Mason, 2020). Target cells of SARS-CoV-2 in other organs have been also identified, e.g. renal tubular epithelial cells and podocytes in the kidney (Chen et al., 2021). Infection of these cells can lead to acute kidney injury (Chen et al., 2021). In some organs, possible target cells of SARS-CoV-2 are still controversial. For instance, COVID-19 patients can exhibit symptoms associated with the central neural system (CNS) like depression and hyposmia. However, the mechanisms of these symptoms are still not clear. In the humanized mouse model and human brain organoid model, neurons in CNS are susceptible to the SARS-CoV-2 virus and their infection can lead to cell death (Song et al., 2021). However, in animal models based on non-human primates, detected SARS-CoV-2 infection in CNS is limited to the epithelium of vasculature but not neurons (Rutkai et al., 2022). For another clinical study, hyposmia associated with COVID-19 is attributed to viral infection of sustentacular cells in olfactory mucosae (Khan et al., 2021). Also, no SARS-CoV-2 infection in olfactory bulbs is detected in samples from patients in this study (Khan et al., 2021).

Normally, infection of viruses to target cells depends on the existence of specific receptors at the cell surface. For HIV-1, the T cell surface receptor CD4 is the major receptor and two chemokine receptors CXCR4 and CCR5 are known coreceptors at the surface of CD4+ T lymphocytes (Berger et al., 1999). And for SARS-CoV-2, angiotensin-converting enzyme 2 (ACE2) has been identified as the major receptor at the surface of AT2 cells (Lan et al., 2020). Hence, a common method to identify viral target cells is to measure the expression level of known viral receptors in different cell types. If a type of cell expresses viral receptors with a high level, it is to be expected that viruses will infect it efficiently. However, this method has several deficiencies. First, it is possible that in addition to known receptors, there are other unidentified receptors that can mediate viral entry into cells. In the example of SARS-CoV-2, neuropilin-1 (Nrp1) has been identified as another viral receptor (Cantuti-Castelvetri et al., 2020). Besides, the existence of receptors is not the only determinant for cells’ susceptibility to a virus. Some viruses can enter cells without specific receptors. For HIV, cell-to-cell contact structures including nanotubes, filopodia and virological synapses can mediate intercellular transmission of viruses (Bracq et al., 2018). Another example is the antibody-dependent enhanced entry (ADE) effect for dengue virus (DENV). During the secondary infection of DENV which belongs to a different serotype, virus-antibody complexes composed of viral particles and non-neutralizing antibodies can bind Fc receptors at the cell surface and mediate viruses’ entry into cells (Flipse et al., 2013). On the other hand, after entering cells, the completion of viral replication cycles requires efficient expression of various protein products encoded by its genome. Hence, the efficiency of virus replication in different cells can be different according to different regulatory mechanisms of gene expression. Regulation of gene expression is a multi-level process including epigenomic, transcriptional, post-transcriptional, translational and post-translational mechanisms. For translational regulatory mechanisms, if viral mRNAs cannot be translated efficiently in a specific cell type, this type of cell can be insensitive to infection of this virus (Stern-Ginossar et al., 2019). In fact, restriction of viral mRNA translation has been reported as a common cellular antiviral mechanism (Stern-Ginossar et al., 2019).

Except for experimental research, biostatistical methods including several statistical indicators have been proposed to evaluate the efficiency of viral mRNA translation. In this paper, for providing indirect evidence for the susceptibility of different human cell types to HIV-1 and SARS-CoV-2, we utilized the codon adaptation index (CAI) as a measure of viral ORFs’ translational efficiency at the translational elongation level in these cell types (SHARP and LI, 1987). This parameter utilizes the codon usage frequency of the concerned RNA and ORFs in a background gene set composed of genes with high expression levels in the concerned biological system (can be a species, an organ or a cell type) as the only evidence to assess the translational efficiency of mRNAs (SHARP and LI, 1987). According to this indicator, the expression efficiency of protein products encoded by the viral genome and the replication ability of the virus in a specific biosystem can be evaluated at the translational level. Currently, most studies that utilized CAI constructed a single high-expression gene set for a species and regarded it as the background gene set mentioned above (Ruiz et al., 2006; Tello et al., 2013; Khandia et al., 2019). This method is appropriate in the research of most prokaryotic organisms like E. Coli and single-cell eukaryotic organisms like yeast, but it is not suitable for assessing the translational efficiency of mRNAs in different cell types of complex multicellular eukaryotic organisms like humans because it does not consider the differences of gene expression patterns in different organs and cell types. For evaluating the translational efficiency of viral mRNAs in different human cell types, the background gene set should be constructed separately in the corresponding cell type. Hereby, in this paper, we selected bulk and single-cell transcriptomic datasets of dozens of human cell types which were reported to be susceptible to HIV-1 and SARS-CoV-2 or locate in major target organs of these viruses according to previous studies. Then we calculated the CAIs of ORFs in genomes of these viruses in these cell types separately and performed downstream analysis. Additionally, we also tried to analyze the effect of viral infection on viral ORFs’ translational efficiency by comparing the CAI between control and infected cells. Finally, we compared the infectious capacity of different kinds of SARS-CoV-2 related coronaviruses in human cells according to the CAIs.

## 2 Methods

### 2.1 Calculation of the codon adaptation index

For a specific RNA in a specific cell type with corresponding high-expression protein-coding (Unless otherwise stated, the term ‘gene’ below only refers to protein-coding genes) gene set, the expression of the original version of CAI while it was proposed (SHARP and LI, 1987) is:


$$CAI={\left(\prod_{k=1}^{L}\frac{p_{k}}{q_{k}}\right)}^{\frac{1}{L}}$$

‘L’ is the number of sense codons (except ATG, the only codon for Met, and TGG, the only codon for Trp) in the ORF of concerned RNA. ‘p_k_’ is the usage frequency of the k-th sense codon (from the initiation codon to the last sense codon before the termination codon) in the ORF of concerned RNA in all synonym codons of the high-expression gene set. ‘q_k_’ is the max usage frequency of the synonym codon of the k-th codon in the ORF of concerned RNA in all synonym codons of the high-expression gene set.

The original version of CAI was used widely to evaluate the translational efficiency of mRNA. However, it has two major problems (Xia, 2007). First, if the usage frequency of a specific codon from the concerned RNA in the high-expression gene set is zero, the CAI of this RNA will be zero regardless of the composition of its other codons (Xia, 2007). Then the function of CAI in evaluating translation efficiency will be lost. For avoiding this condition, we can convert p_k_ and q_k_ in the CAI expression to p_k_+0.01 and q_k_+0.01. Second, for three kinds of amino acids with six corresponding codons (Ser, Arg and Leu), their codons can be divided into two smaller groups according to the first two bases. In these conditions, the same amino acid coded by different groups of codons can be considered as different amino acids in the calculation of CAI (Xia, 2007). For the CAI calculation in our study, both two improvements mentioned above were adopted.

### 2.2 Constructing the high-expression gene set of each concerned cell type

#### 2.2.1 Collection of the human RNA-seq data of concerned cell types.

For constructing high-expression gene sets, measuring the expression level of genes in different cell types is necessary. Transcriptomic techniques, e.g. bulk and single-cell RNA-seq, are the main methods to measure gene expression levels in tissues or cells currently (Stark et al., 2019). In our study, human RNA-seq datasets of different cell types (shown in **Table 1**) from the NCBI GEO database (https://www.ncbi.nlm.nih.gov/geo/ ) were selected for constructing corresponding datasets. The detailed process for selecting datasets is mentioned in Supplemental Methods (Li et al., 2009; Liao et al., 2014; Kim et al., 2019). Finally, 19 bulk RNA-seq datasets (including 2 datasets with corresponding Ribo-seq data) corresponding to different types of cells and 2 single-cell RNA-seq datasets corresponding to major target ‘organs’ of SARS-CoV-2 and HIV-1 (lung and PBMC respectively) were selected.

**Table 1:** Concerned human cell types and corresponding RNA-seq datasets in this study.

Groups	Cell types	GSE ID of corresponding datasets analyzed in this study	Types
Bulk RNA-seq datasets of unstimulated human monocyte-macrophage system	Blood monocytes	GSE159249 (Morante-Palacios et al., 2021)	Bulk RNA-seq
	Dermal macrophages	GSE166639 (Rhodes et al., 2021)	Bulk RNA-seq
	I+ dermal dendritic cells	GSE166639 (Rhodes et al., 2021)	Bulk RNA-seq
	I- dermal dendritic cells	GSE166639 (Rhodes et al., 2021)	Bulk RNA-seq
	Langerhans cells	GSE166639 (Rhodes et al., 2021)	Bulk RNA-seq
	*In vitro* differentiated dendritic cells	GSE166639 (Rhodes et al., 2021)	Bulk RNA-seq
	*In vitro* differentiated macrophages	GSE193336 (Li et al., 2022)	Bulk RNA-seq
	*In vitro* differentiated osteoclasts	GSE166535 (Larrouture et al., 2021)	Bulk RNA-seq
	Kupffer cells (Hepatic macrophages)	GSE123661	Bulk RNA-seq
	Colonic macrophages	GSE124350	Bulk RNA-seq
	Microgolias from occipital cortex	GSE111972 (van der Poel et al., 2019)	Bulk RNA-seq
	Microgolias from corpus callosum	GSE111972 (van der Poel et al., 2019)	Bulk RNA-seq
Bulk RNA-seq datasets of subtypes of unstimulated human CD4+ T lymphocytes	Naive CD4+ T lymphocytes	GSE179613 (Giles et al., 2022)	Bulk RNA-seq
	Nonnaive CD4+ T lymphocytes	GSE179613 (Giles et al., 2022)	Bulk RNA-seq
	Tfh CD4+ T lymphocytes	GSE179613 (Giles et al., 2022)	Bulk RNA-seq
	Treg CD4+ T lymphocytes	GSE179613 (Giles et al., 2022)	Bulk RNA-seq
Bulk RNA-seq datasets of subtypes of unstimulated reported SARS-CoV-2 target and untargeted cells in human kidney	Podocytes	GSE185292 (Ren et al., 2021)	Bulk RNA-seq
	Mesangial cells	GSE185293 (Ren et al., 2021)	Bulk RNA-seq
Bulk RNA-seq datasets of unstimulated major cell types in key metabolic organs (liver and adipose tissue)	Hepatocytes	GSE201169 (Sullivan et al., 2021)	Bulk RNA-seq
	CD133+ cholangiocytes	GSE155498 (Hallett et al., 2022)	Bulk RNA-seq
	CD133- cholangiocytes	GSE155498 (Hallett et al., 2022)	Bulk RNA-seq
	Hepatic satellite cells	GSE179395 (Gart et al., 2021)	Bulk RNA-seq
	Adipocytes	GSE201908 (Friesen et al., 2022)	Bulk RNA-seq
Smart-seq2 based single-cell RNA-seq dataset of human lung	16 Cell types: AT1 epithelium, AT2 epithelium, B cells, CD4+ T cells, CD8 T cells, Ciliated cells, Dendritic cells, Endothelial cells, Fibroblasts, Granulocytes, macrophages, Mast cells, Monocytes, NK cells, Other epithelial cells, Other T cells 3 large groups: Epithelial cells, T cells, Myeloid cells	No GSE ID; BioProject: PRJNA591860 (Maynard et al., 2020)	Single-cell RNA-seq
Smart-seq2 based single-cell RNA-seq dataset of human peripheral blood mononuclear cells (PBMCs)	7 Cell types: B cells, CD4+ T cells, CD8+ T cells, CD14+ monocytes, CD16+ monocytes, NK cells, platelets	GSE132044 (Deng et al., 2020)	Single-cell RNA-seq
Bulk RNA-seq datasets of infected or cytokine stimulated and control human cells	HEK293T-hACE2 cell, control and infected with SARS-CoV-2	GSE169158 (Sun et al., 2021)	Bulk RNA-seq
	A549 cell, control and infected with SARS-CoV-2	GSE147507 (Daamen et al., 2021)	Bulk RNA-seq
	A549-hACE2 cell, control and infected with SARS-CoV-2	GSE147507 (Daamen et al., 2021)	Bulk RNA-seq
	Calu3 cell, control and infected with SARS-CoV-2	GSE147507 (Daamen et al., 2021)	Bulk RNA-seq
	NHBE cell, control and infected with SARS-CoV-2	GSE147507 (Daamen et al., 2021)	Bulk RNA-seq
	Organoid formed by primary human lung epithelium, control and infected with SARS-CoV-2	GSE160435 (Mulay et al., 2021)	Bulk RNA-seq
	Human primary proximal tubule (HPPT) cells were stimulated with IFNα, IFNβ, IFNγ and IL-1β	GSE161916 (Jankowski et al., 2021)	Bulk RNA-seq
Bulk RNA-seq and paired Ribo-seq datasets of infected or control human cells	Volunteer-derived primary CD4+ T cells. Control (cultured for 4 and 96h) and infected with HIV-1 (for 4 and 96h)	GSE158930 (Puray-Chavez et al., 2022)	Bulk RNA-seq and Ribo-seq
	HBEC cells, control (cultured for 4 and 96h) and infected with SARS-CoV-2 (for 4, 24, 48, 72, 96h)	GSE158930 (Puray-Chavez et al., 2022)	Bulk RNA-seq and Ribo-seq

#### 2.2.2 Gene-level expression analysis of RNA-seq datasets

To determine gene expression levels by RNA-seq, a series of processes are needed to convert raw sequencing data into a normalized gene-level expression matrix (FPKM). The pipeline of this process is mentioned in Supplemental Methods. (Li et al., 2009; Liao et al., 2014; []Kim et al., 2019) For single-cell RNA-seq data, the R package Seurat (version: 4.1.1) was used to preprocess, cluster and visualize cells according to their gene expression patterns. Cell types were annotated according to the corresponding literature of the dataset. Only cell types with more than ten cells in each single-cell RNA-seq dataset were used for CAI calculation and further analysis.

Gene-level expression data (raw counts and FPKM) for all RNA-seq datasets used in this study is provided in Github (https://github.com/Renruohan/CAIvirus). For two single-cell RNA-seq datasets, the corresponding relationship between cells and cell types/large groups was also listed in Github. Also, intermediate results of single-cell RNA-seq datasets by Seurat including cell clustering and visualization in low-dimension space, ratio or number of cells belonging to each cell type/large group and markers to identify each cell type/large group were shown in **Supplemental Figures 1**, **2**.

#### 2.2.3 Select high-expression genes in each concerned cell type

According to the concept of CAI, the definition of the high-expression gene set is arbitrary. In previous studies, typical housekeeping genes like ribosomal genes and histone genes are usually utilized to construct the high-expression gene set. The lack of cell type-specificity made this strategy unsuitable for our study. For highlighting differences in gene expression patterns in various cell types, we selected the top 200 protein-coding genes (contain both housekeeping genes and genes with tissue/cell-specific expression) with the highest mean FPKM level in biological repetitions (for bulk RNA-seq datasets) or cells (for single-cell RNA-seq datasets) for a cell type to construct the high-expression gene set of this cell type. Also, we found that expression levels of some genes changed significantly in different biological repetitions or cells of the same cell type. Hence, before selecting high-expression genes as mentioned above, we removed genes whose standard deviations of the expression levels are large than the average value. The selected top 200 high-expression gene sets and their normalized expression level (FPKM) for all RNA-seq datasets used in this study are provided in **Supplemental File 1**. To characterize the functional characteristics of high-expression gene sets in different cell types, we performed GO-BP and KEGG enrichment analysis on them by R package clusterProfiler (Wu et al., 2021) (version: 4.0.5) and Org.Hs.eg.db (version: 3.13.0). Results of enrichment analysis for three representative datasets (bloodmonocytes, CD4+ Tfh in bulk RNA-seq datasets and dendritic cells in scRNA-seq datasets) are provided in **Supplemental File 2**.

#### 2.2.4 Alternative splicing analysis of the high-expression genes

Different from prokaryotic genes, mRNAs transcripted from eukaryotic genes usually experience complex processing including splicing. Because of the existence of alternative splicing, the same mRNA precursor can be processed to different mature mRNAs and translated to different protein products. As a result, it is necessary to discriminate different splicing isoforms of genes in the high-expression gene set. The process of calculating the approximate expression level of splicing isoforms of high-expression genes is mentioned in Supplemental Methods. For each gene, only the isoform with the highest approximate expression level of each gene was utilized to construct the codon usage frequency table of the high-expression gene set, then p_k_ and q_k_ in this expression of CAI could be acquired from this table. CDS sequences of the isoform with the highest approximate FPKM of each gene in high-expression gene sets for all RNA-seq datasets used in this study are provided in **Supplemental File 3**.

#### 2.2.5 Verifying the relationship between CAI and translational efficiency of endogenous genes

For verifying if CAI can represent genes’ translational efficiency, we calculated the CAI of the top 5000 highly expressed genes (except genes that are not detected in Ribo-seq experiments) in two groups of paired RNA-seq and Ribo-seq datasets of control cells and cells infected with HIV-1 or SARS-CoV-2 for a certain time. Also, we calculated the translational efficiency by dividing their normalized expression values (FPKM) in Ribo-seq datasets by values in RNA-seq datasets of these genes and then took the logarithm. Then we performed a linear regression analysis between genes’ CAI and translational efficiency in each cell type. Furthermore, for verifying if the CAI of endogenous genes calculated by cell type-specific background gene sets correlated better with translational efficiency than CAI based on commonly used nonspecific background gene sets, we constructed a nonspecific background gene set reference to previously mentioned methods. First, typical housekeeping genes-ribosomal protein genes were selected. Then, we filtered them according to expression patterns, preserving only the ribosomal genes that were present in the top 200 highly expressed genes of at least one cell type (except cells infected by virus or stimulated by cytokines and paired control groups in the same dataset). The CAI of endogenous genes based on this nonspecific background gene set and the correlation between this ‘nonspecific’ CAI and translational efficiency is calculated in the same way as above. In CAI calculation, for each gene in the nonspecific background gene set, one isoform from all isoforms that appear in the top 200 highly expressed gene set of at least one cell type was selected randomly and utilized in the construction of the codon usage frequency table.

Data processing for Ribo-seq datasets is mentioned in Supplemental Methods (Dobin et al., 2012).

Gene-level expression data (raw counts and FPKM) for all Ribo-seq datasets used in this study is provided on the Github page. The CAIs and the translational efficiency of the top 5000 highly-expressed genes for all RNA-seq/Ribo-seq pair datasets in this study are provided in **Supplemental File 4**.

### 2.3 Collecting genomic and ORF sequences of HIV-1, SARS-CoV-2 and other coronaviruses

For HIV-1, genomic sequences were collected from the HIV sequence database (Kuiken et al., 2003) (https://www.hiv.lanl.gov/content/sequence/HIV/mainpage.html). There are 21 known subtypes (A1, A2, A3, A4, A6, A7, A8, B, C, D, F1, F2, G, H, J, K, L, N, O, P, U) for HIV-1. Because all genome sequences of subtype K in the database do not meet our standards, this subtype of viruses was not collected and included in the analysis below. For every other subtype, 2-3 genomic sequences were collected to remove random bias. Finally, 58 genomic sequences of HIV-1 were collected for further analysis. Because some collected HIV-1 genomic sequences do not contain all functional ORFs, we exclude some ‘problematic’ ORFs in CAI calculation and further analysis (the detailed process is mentioned in Supplemental Methods). Major information (patient ID, accession ID, name, subtype, country and year for separation, completeness and length for genomic sequence and annotation for specific conditions) and ORFs’ sequences for all 58 HIV-1 strains used in this study are provided in **Supplemental File 5**. If one ORF is regarded as ‘problematic’ according to the above standards and excluded in CAI calculation, this ORF will be marked as “nonfunction” in the table.

For SARS-CoV-2, the genomic sequence of the original strain in Wuhan was collected from the NCBI nucleotide database (https://www.ncbi.nlm.nih.gov/nuccore ) and five variants of concerns (Alpha, Beta, Gamma, Delta and Omicron) identified by the World Health Organization (WHO) were collected from GISAID influenza virus and SARS-CoV-2 sequences database (https://www.gisaid.org ). For each variant of concern, 5 genomic sequences were collected to remove random bias. There are six ORFs for essential primary protein products (ORF1a, ORF1ab, S, E, M, N. The relationship between ORF1ab and ORF1a is similar to the relationship between gag-pol and gag in the example of HIV-1) and six ORFs for non-essential primary protein products (ORF3a, ORF6, ORF7a, ORF7b, ORF8, ORF10) in SARS-CoV-2 genomes (Al-Qaaneh et al., 2021b). Although the deficiency of non-essential ORFs is common in genomes of some SARS-CoV-2 strains, all ORFs are complete and functional in SARS-CoV-2 genomic sequences collected by our studies. Therefore, all twelve ORFs of these sequences are involved in downstream analysis. Major information (accession ID, name, VOCs, country and time for separation) and ORFs’ sequences for all 26 SARS-CoV-2 strains used in this study are provided in **Supplemental File 6**.

For six other coronavirus species (SARS-CoV, MERS-CoV, HCoV-OC43, HCoV-NL63, HCoV-HKU1, HCoV-229E) that can infect humans, genomic sequences were collected from the NCBI nucleotide database. For each species, only the reference genomic sequence was collected. For each of these sequences, all ORFs annotated in corresponding species are functional and are involved in downstream analysis. ORFs’ sequences for all six other coronaviruses used in this study are provided in **Supplemental File 7**.

### 2.4 Calculation and downstream analysis of viral ORFs’ CAI

After sequence collection, the CAI of ORFs of all HIV-1, SARS-CoV-2 and other coronavirus strains above was calculated according to Part 1 in Methods. Results for three groups of viruses are provided in **Supplemental Files 8-10** separately. The relative usage frequency of synonymous codons (relative synonymous codon usage, RSCU) of the top 200 high-expressed genes in all cell types which is needed for CAI calculation is in **Supplemental File 11**.

For HIV-1, we compared the CAIs of their ORFs in several groups of different cell types (1) 16 cell types and 3 large groups in the lung. (2) 7 cell types of peripheral blood mononuclear cells (PBMC) (3) 12 cell types of the monocyte-macrophage system (4) 4 subtypes of CD4+ T lymphocytes (5) 5 cell types from two key metabolic organs. To test if the CAI can reflect the evolutionary relationship of different viral strains, hierarchical clustering algorithms were utilized to cluster HIV-1 viral strains based on their ORFs’ standardized (z-score) CAIs in different cell types. For visualizing the results of our analysis, the R package ggplot2 (version: 3.3.6) was utilized.

For SARS-CoV-2 subtypes and six other coronavirus species, we compared the CAIs of their ORFs in the lung dataset (16 cell types and 3 large groups in the lung). We also showed the CAI distribution of SARS-CoV-2 in glomerulus-podocytes and mesangial cells because the kidney was reported as an affected organ of COVID-19 (Chen et al., 2021). In addition, we hoped to explore the change of CAI to observe the change of cell state after viral infection. Therefore, we also calculated the CAIs of SARS-CoV-2 ORFs in the Bulk RNA-seq datasets of infected and control human cells. For visualizing the results of our analysis, the R package ggplot2 (version: 3.3.6) was utilized.

## 3 Results

### 3.1 Descriptive analysis of endogenous genes: Enrichment analysis, CAI distribution and quantitative relationship between CAI and translational efficiency

**Figure 1:**
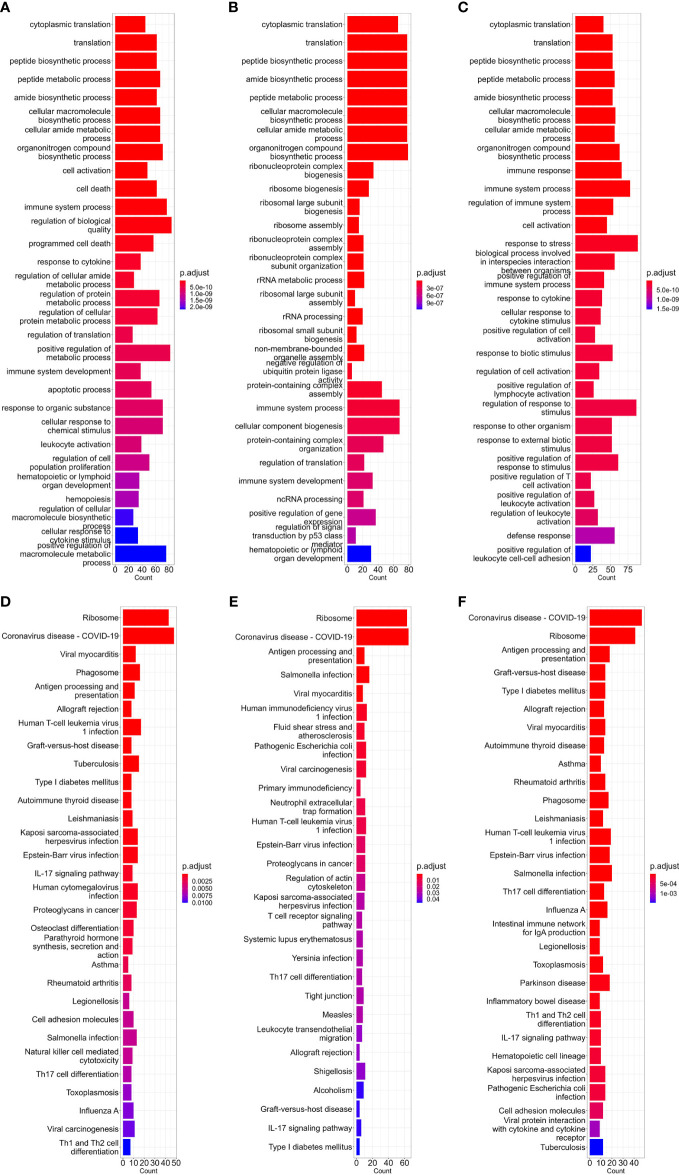
Barplot of GO-BP **(A–C)** and KEGG **(D–F)** enrichment analysis results of bloodmonocytes **(A, D)**, CD4+ Tfh **(B, E)** in bulk RNA-seq datasets and dendritic cells **(C, F)** in lung scRNA-seq datasets. For each analysis, the top 30 enriched pathways according to p-value are shown.

The first part of our study was constructing high-expression gene sets for different cell types. As discussed in the ‘Methods’ part above, for comparing the translational efficiency of viruses in different cell types, except for housekeeping high-expression genes (e.g. ribosomal protein genes), these gene sets should include enough cell type-specific genes which can represent unique gene expression patterns of these cell types. To verify if the high-expression gene sets we constructed met these conditions, we performed GO-BP (biological pathway) and KEGG enrichment analysis which can reflect which biological processes these genes are involved in. Results of the dataset corresponding to three types of cells (bloodmonocyte (Morante-Palacios et al., 2021), CD4+ Tfh (Giles et al., 2022) in bulk RNA datasets and dendritic cells in lung scRNA-seq datasets (Maynard et al., 2020)) are exhibited (**Figures 1A-F**). According to these results, except for ribosome or cytosolic translation-related pathways, several pathways related to immune response (e.g. immune system process in BP or COVID-19 and other infectious diseases in KEGG) which is the major function of these cell types are also significantly enriched. In conclusion, high-expression gene sets constructed for each concerned cell type in this study met our requirement.

**Figure 2:**
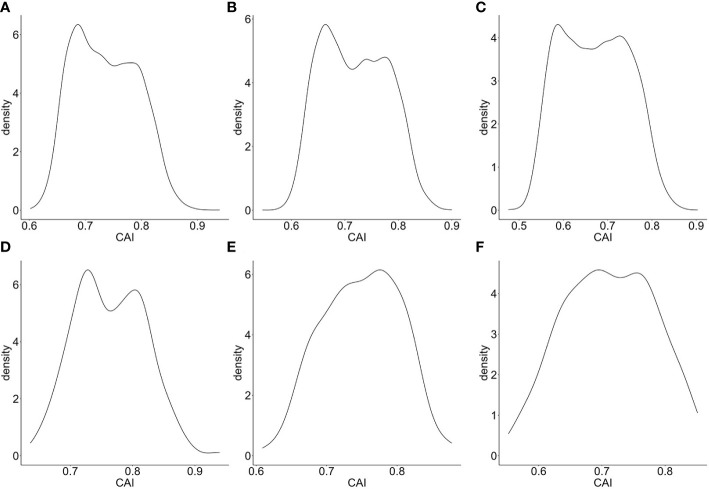
Density plot for the distribution of CAIs in the top 5000 **(A–C)**/200 **(D–F)** highly expressed genes of bulk RNA-seq datasets corresponding to CD4+ Tfh **(A, D)**, Kupffer cells **(B, E)** and Langerhans cells **(C, F)**.

Also, we calculated the CAIs of the top 5000 highly expressed genes in bulk RNA-seq datasets corresponding to CD4+ Tfh (Giles et al., 2022), Kupffer cells and Langerhans cells (Rhodes et al., 2021). The distributions of the CAIs of the top 5000 and the top 200 highly expressed genes in these cells are shown in **Figure 2**. Among these cell types, the CAI of most endogenous genes is mainly between 0.6-0.85. Also, the mean CAI of the top 5000 or the 200 genes is separately 0.736/0.763, 0.714/0.744, 0.664/0.697 in three types of cells. This result reveals significant differences in CAI distribution for endogenous genes in different cell types: the overall trend of endogenous genes’ CAIs in Langerhans cells is significantly lower than in the other two types of cells. On the other hand, we found there is a statistically significant but slightly positive correlation (r=0.102, 0.0763, 0.0468; p=5.10e-13, 6.48e-08, 0.000939) between gene expression levels (FPKM value) and CAIs in the top 5000 highly expressed genes of all three cell types. These results may reflect the complex relationship between translational efficiency and mRNA expression level.

**Figure 3:**
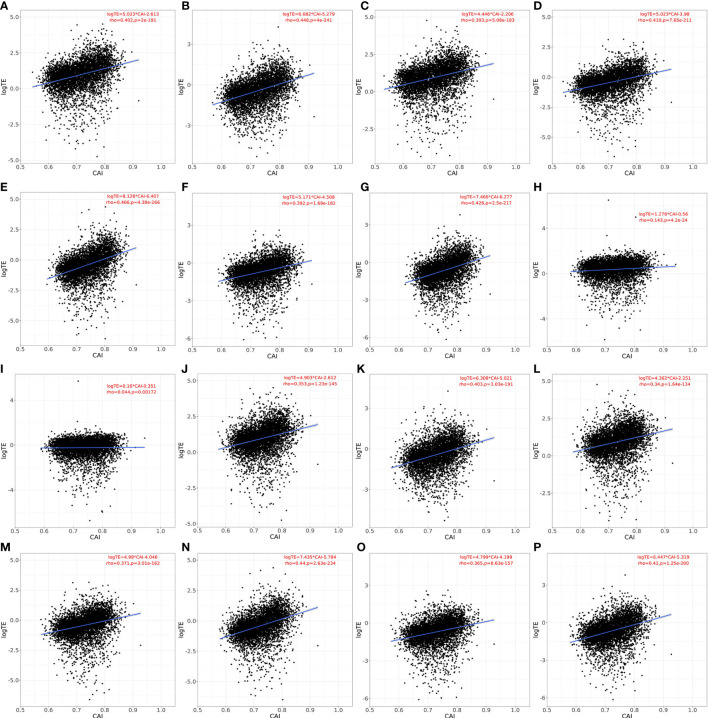
Linear relationship, spearman correlation coefficient and corresponding p-value between CAI and translational efficiency for the top 5000 highly expressed genes (except genes which are not detected in Ribo-seq experiments) in each paired RNA-seq and Ribo-seq datasets. a-g. HBEC cells (cultured for 4 or 96h for **A, B**; infected with SARS-CoV-2 for 4, 24, 48, 72, 96h for **C-G**), normal CAI (cell-specific highly expressed gene set as the background gene set); **H, I**. patient-derived primary CD4+ T cells (control for **H**; infected with HIV-1 for **I**), normal CAI (cell-specific highly expressed gene set as the background gene set); **J-P**. HBEC cells (cultured for 4 or 96h for **J, K**; infected with SARS-CoV-2 for 4, 24, 48, 72, 96h for **L-P**), ‘nonspecific’ CAI (constructed nonspecific gene set as the background gene set).

As mentioned in the introduction part, the regulatory mechanism of cellular translation is a complex process. Both the initialization and elongation of translation are tightly regulated to maintain the normal physiological activity of cells. However, CAI or similar indicators based on codon adaptation can only reflect the regulation at the translational elongation level. Thus, the effectiveness of CAI in evaluating translational efficiency still needs to be verified. For establishing the relationship between CAI and translational efficiency and for verifying the rationality of representing genes’ translational efficiency through calculating CAI, we perform the linear regression analysis between CAI and translational efficiency for the top 5000 highly expressed genes in each paired RNA-seq and Ribo-seq datasets. Results are shown in **Figure 3**. In Human Bronchial Epithelial Cells (HBEC) and patient-derived primary CD4+ T cells without viral infection, there was a significant positive correlation between CAI and translational efficiency of mRNAs regardless of viral infection or cultured time. In patient-derived primary CD4+ T cells infected with HIV, the correlation between CAI and translational efficiency of mRNA is significantly slightly. This phenomenon can be attributed to the modulation of the tRNA pool in host cells by HIV-1 infection (Anna et al., 2011). The rationality of CAI is based on the hypothesis that highly expressed genes are optimized at the codon level and translated with high efficiency. However, this assumption is true only when the endogenic tRNA pools are utilized for translation. If the tRNA pools are modulated by viruses, cellular preference for codons in translational elongation will change. Then the translational efficiency of highly expressed genes may decrease and the relationship between CAI and translational efficiency will be decoupled. Because HIV-1 can manipulate cellular translational regulatory machinery by modulating tRNA pools, we can postulate CAI of HIV-1 ORFs mainly reflects the potency of the establishment but not the maintenance of HIV-1 infection. On the other hand, infection of SARS-CoV-2 does not weaken the correlation between CAI and translational efficiency significantly, which implies its effect on cellular translational regulatory machinery is weaker. Thus, we can postulate that the CAI of ORFs can reflect the potency of both the establishment and the maintenance of SARS-CoV-2 infection.

An important feature of our study was to calculate CAI according to cell type-specific background gene sets. For verifying the advantages of this strategy, we constructed nonspecific background gene sets. The detailed process of constructing can be seen in the method above. According to this gene set, we calculated the CAI of the top 5000 high-expression coding genes in previously mentioned RNA-seq datasets of HBEC with or without SARS-CoV-2 infection and analyzed the correlation between CAI and translational efficiency subsequently. The results are shown in **Figure 3**. We found that for each condition with paired RNA-seq/Ribo-seq dataset, compared to CAI calculated by cell type-specific background gene sets (**Figures 3A-G**), the translational efficiency of endogenous genes exhibits a lower correlation coefficient and decreased significance (larger p-value) with CAI calculated by nonspecific background gene sets (**Figures 3J-P**). For example, the correlation coefficient (rho-value) in **Figure 3J** (showing HBEC cells cultured for 4h; ‘nonspecific’ CAI) is lower than that in **Figure 3A** (showing HBEC cells cultured for 4h; ‘cell type-specific’ CAI), while the p-value in **Figure 3J** is much larger than that in **Figure 3A**. Thus, at least for endogenous genes, cell type-specific CAI is a better indicator for assessing translational efficiency and the advantage of our method is verified.

### 3.2 CAI of HIV-1 virus

#### 3.2.1 General situation of HIV-1 ORFs’ CAIs

**Figure 4:**
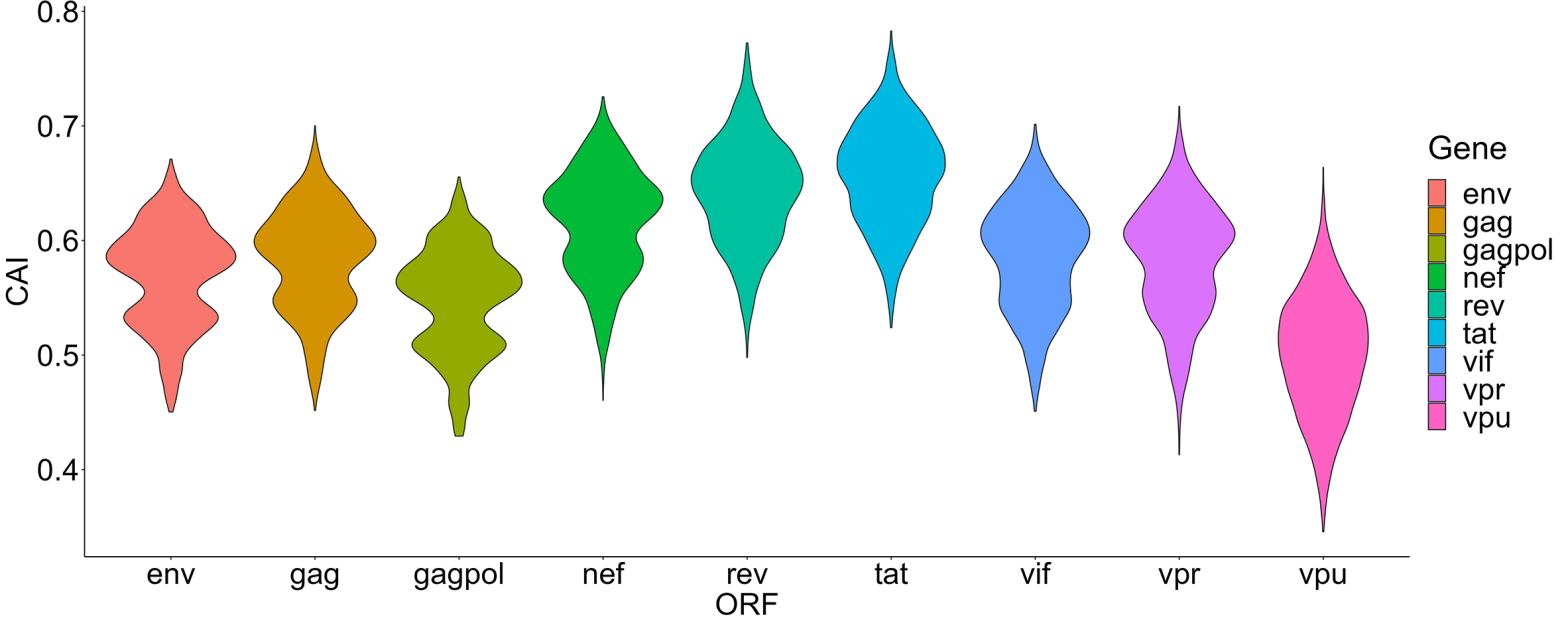
The overall CAI value distribution of different HIV-1 ORFs in all unstimulated cell types/subtypes/large groups from bulk RNA-seq datasets and scRNA-seq datasets shown by violin plot. Each violin shows the overall CAI value distribution of a specific HIV-1 ORF in all cell types/subtypes/large groups (containing genomes of 58 HIV-1 strains belonging to 20 subtypes).

In this section, we mainly focus on the CAIs of HIV-1 in different human cell types. We calculated the CAIs of ORFs for 58 HIV-1 genomic sequences in all collected unstimulated cell types/subtypes/large groups (some cell types like monocytes can be repetitive between bulk and single-cell RNA-seq datasets) according to the previous mentioned bulk RNA-seq datasets and scRNA-seq datasets. As discussed earlier, because HIV-1 can modulate cellular tRNA pools and improve the translational efficiency of its ORFs (Anna et al., 2011), results in this part mainly reflect the capacity of the establishment but not the maintenance of HIV-1 infection. **Figure 4** shows the overall CAI distribution of ORFs from all collected HIV-1 sequences in all studied cell types (from either bulk RNA-seq or scRNA-seq datasets). For all analyzed strains, the CAI of a single ORF varies from 0.346 to 0.765. The average CAI among strains and cell types for each ORF fluctuates from 0.500 (vpu) to 0.658 (tat). ORFs tat and rev have the highest CAI, which is consistent with a previous study (Supinya et al., 2018). Compared to the CAIs of endogenous genes exhibited in **Figure 2**, in general, HIV-1 ORFs exhibited significantly lower CAI than high-expressed endogenous genes. Results of CAI calculation based on scRNA-seq datasets (see below) are also consistent with this point. As a result, we can postulate that the translational efficiency of ORFs in the HIV-1 genome is lower than most endogenous genes and HIV-1 has been still not well adapted to translation-level regulatory mechanisms of gene expression in unstimulated human cells, at least in cell types we studied. This result may explain why HIV-1 should modulate the cellular tRNA pool to improve its ORFs’ translational efficiency (Anna et al., 2011).

#### 3.2.2 Comparing HIV-1 ORFs’ CAIs in different cell types

**Figure 5:**
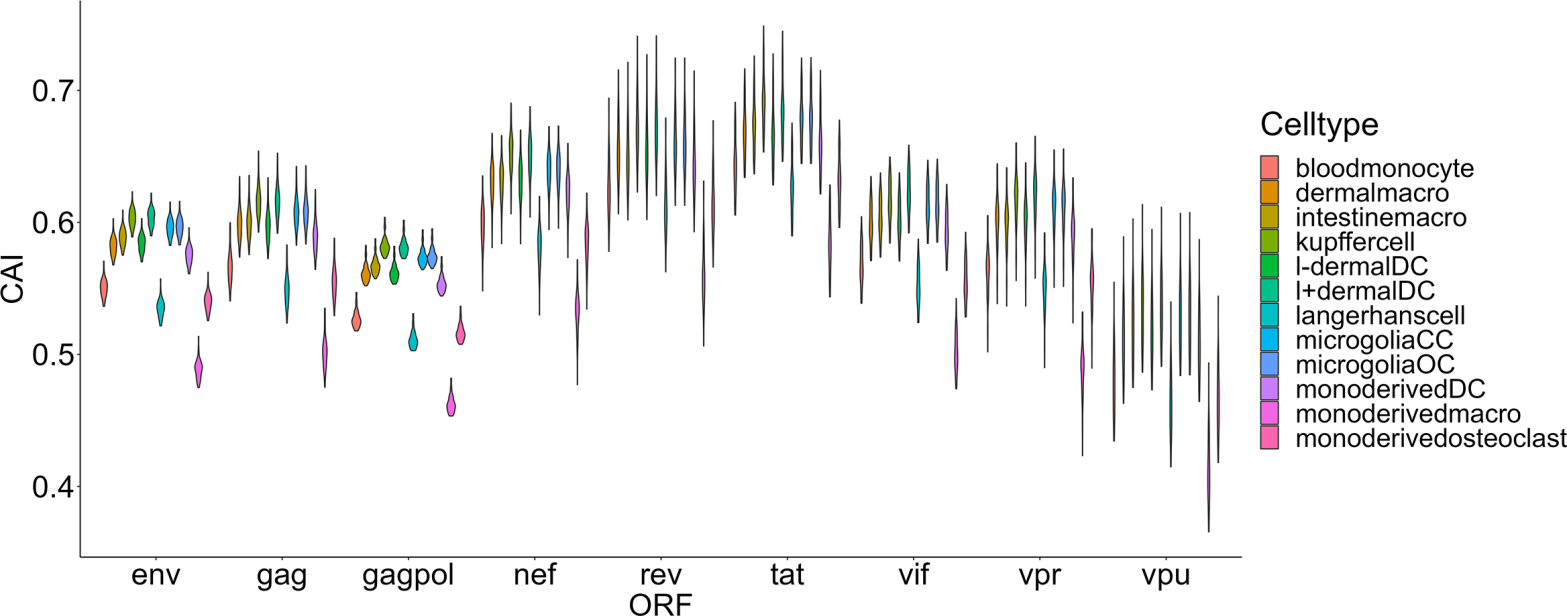
The CAI value distribution of different HIV-1 ORFs in each unstimulated cell type belonging to the monocyte-macrophage system from bulk RNA-seq datasets shown by violin plot. Each violin shows the CAI value distribution of a specific HIV-1 ORF in a specific cell type.

The damage of viruses to cells can be comprehensively measured from two aspects, one is their ability to enter cells, and the other is their destructive ability after entering cells. Thus, we also hoped to use the CAI to indirectly reflect the adaptation of the virus to the translation system of the host cell and the ability of the virus to express proteins and damage host cells after entering. We compared HIV-1 ORFs’ CAIs in several groups of cell types. First, we analyzed HIV-1 ORFs’ CAIs in several types of monocyte-macrophage system cells from bulk RNA-seq data (**Figure 5**). Among these cell types, the highest CAI appears in Kupffer cells (hepatic macrophages) and I+ dendritic cells. On the other hand, the lowest CAI appears in macrophages differentiated from monocytes *in vitro*. The second lowest CAI appears in Langerhans cells which are tissue-resident specialized macrophages of the skin. However, it has been reported that *in vitro* differentiated macrophage is susceptible to HIV-1 and *in vitro* HIV-1 infection models based on this cell type have been established (Deshiere et al., 2017). Also, during sexual transmission, Langerhans cells are one of the first groups of HIV-1 target cells and their migrations to lymphatic nodes mediate viruses’ infection to CD4+ T cells (Botting et al., 2017). Hence, this result is not consistent with the actual pathogenesis of HIV-1 infection, which provides evidence for the decoupling of CAI and translational efficiency by HIV-1 continuous infection. In addition, according to **Figure 5**, we found that the variation of CAI of three ORFs for essential primary protein products (gag, gag-pol and env) is significantly lower than the other six ORFs for non-essential primary protein products (nef, rev, tat, vif, vpr and vpu). This result can be attributed to different mutation rates of genes that encode essential and non-essential protein products in HIV genomes.

**Figure 6:**
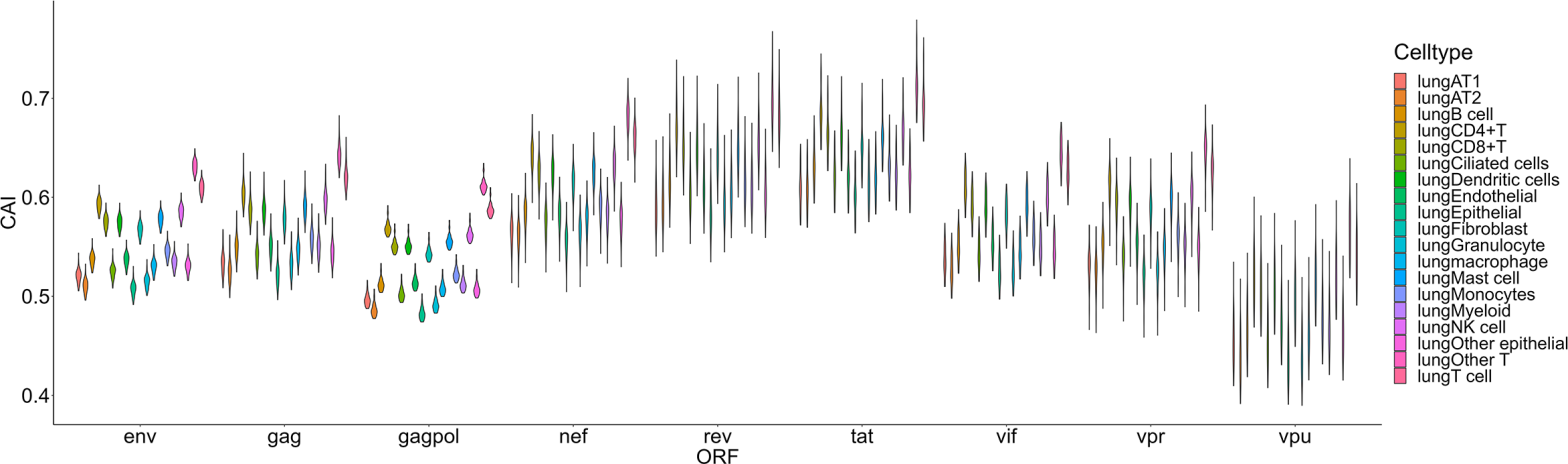
The CAI value distribution of different HIV-1 ORFs in each cell type/large group from the Smart-seq2 based lung scRNA-seq dataset shown by violin plot. Each violin shows the CAI value distribution of a specific HIV-1 ORF in a cell type/large group.

The second group we analyzed is a Smart-seq2 based single-cell RNA-seq dataset of the lung (Maynard et al., 2020) which contains 16 types and 3 large groups of cells as mentioned in the ‘Methods’ part (**Figure 6**). In this dataset, the highest overall CAI of HIV-1 ORFs appears in the group of ‘other T cells’. This group refers to T cells with no detected expression of CD4 and CD8. According to the result of Louvain clustering algorithms performed by Seurat, this group locates in the same group with CD4+ T cells. Hereby, most cells in this group are likely CD4+ T cells whose CD4 expression was not detected for some technical reasons. On the other hand, the CAI of HIV ORFs in all T cells is the second highest and in CD4+ T cells is the third highest among all cell types and large groups. These results are consistent with the conventional opinion that CD4+ T cells are major host cells of HIV-1 in humans. On the other hand, several types of epithelial cells which are not major HIV-1 target cells exhibit relatively low CAIs. However, the CAI of another well-studied target type of cells of HIV-1 (macrophages) is similar to epithelial cells. Finally, it should be noted that different cell types belonging to the hematopoietic lineage exhibit significant variance in HIV-1 ORFs’ CAI. Thus, two cell types originating from the same developmental lineage do not mean that HIV-1 exhibits similar ORFs’ CAI in them.

**Figure 7:**
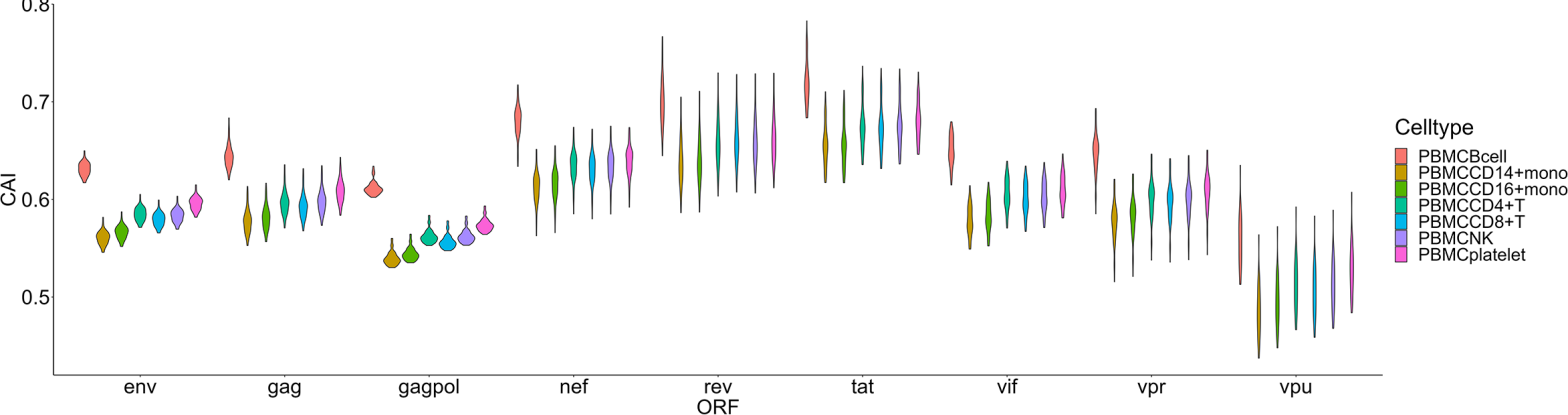
The CAI value distribution of different HIV-1 ORFs in each cell type from the Smart-seq2 based PBMC scRNA-seq dataset shown by violin plot. Each violin shows the CAI value distribution of a specific HIV-1 ORF in a cell type.

For another single-cell RNA-seq dataset of PBMC (Deng et al., 2020), postulated susceptibility of cell types based on CAI is also not consistent with previous experiment studies (**Figure 7**). For HIV-1 ORFs of various strains, their mean CAIs in B cells but not CD4+ or other subtypes of T cells are highest. Also, in two subtypes of monocyte in the blood which have been reported to be host cells of HIV-1, HIV-1 ORFs exhibit the lowest CAI in all cell types. These results imply that CAI is not a good indicator for evaluating cell types’ susceptibility to HIV-1 in peripheral blood.

**Figure 8:**
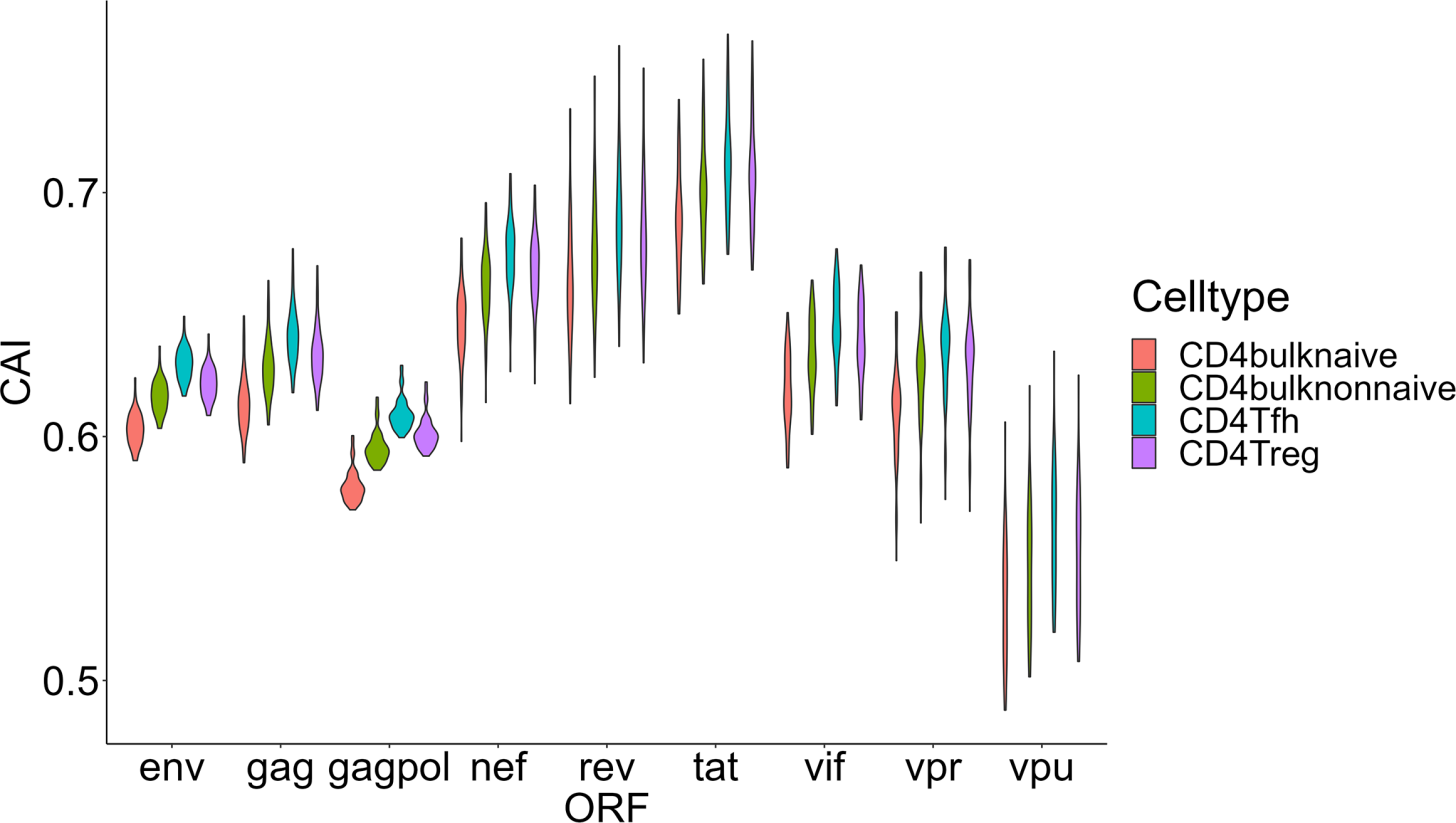
The CAI value distribution of different HIV-1 ORFs in each unstimulated cell subtype of CD4+ T lymphocytes from bulk RNA-seq datasets shown by violin plot. Each violin shows the CAI value distribution of a specific HIV-1 ORF in a specific CD4+ T lymphocyte subtype.

Then we compared HIV-1 ORFs’ CAIs in four different subtypes of CD4+ T cells in peripheral blood (**Figure 8**). In this comparison, the overall trend of CAIs for HIV-1 ORFs in these subtypes is Tfh>Treg> CD4+ T nonnaive> CD4+ T naive. The highest HIV-1 ORFs’ CAI in Tfh is consistent with several recent studies which identify Tfh as a highly susceptible subtype for HIV-1 and a major site for HIV-1 replication and production during untreated infection (Perreau et al., 2013). However, it should be noted that the differences in HIV-1 ORFs’ CAI between these subtypes are small (<0.03 for the average CAI of each ORF in different subtypes, corresponding translational efficiency differences less than 1.04 times according to the linear relationship between CAI and translational efficiency of endogenous genes in patient-derived CD4+ T cells from **Figure 3H**). In conclusion, these results show that CAI can be used as a reference but is not conclusive evidence to evaluate cells’ susceptibility to HIV-1.

**Figure 9:**
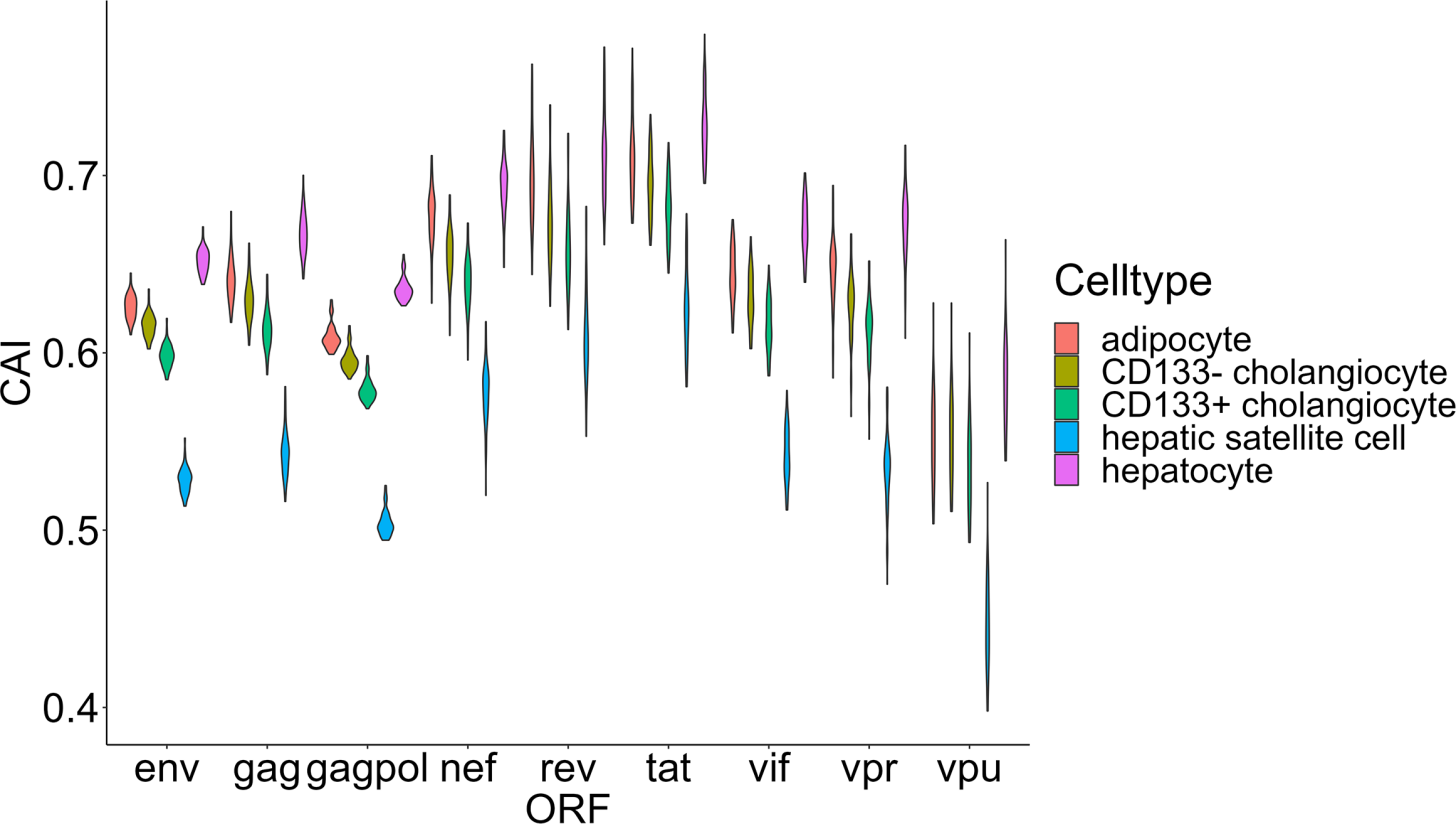
The CAI value distribution of different HIV-1 ORFs in four major cell types (adipocyte, hepatocytes, CD133+/CD133- cholangiocytes and hepatic satellite cells) from two key metabolic organs (liver and adipose tissue) from bulk RNA-seq datasets shown by violin plot. Each violin shows the CAI value distribution of a specific HIV-1 ORF in a specific cell type.

Finally, we focused on two organs that play important roles in metabolic regulation-liver and adipose tissue. We selected four types of nonimmune cells (hepatocytes, CD133+ and CD133- cholangiocytes and hepatic satellite cells) in the liver and adipocytes (the major type of cell that stores lipids in adipose tissue) for CAI calculation and further analysis (**Figure 9**). Some studies have explored the role of these key metabolic organs in the pathology of AIDS. For adipose tissue, its dysfunction related to HIV-1 infection is common in patients with AIDS (Koethe, 2017) and it has been proposed as a reservoir for HIV-1 (Couturier and Lewis, 2018). Nevertheless, direct infection of HIV-1 to adipocytes has been not reported (Couturier and Lewis, 2018). For the liver, the susceptibility of its components to HIV-1 has been studied. Both *in vivo* and *in vitro* studies have shown the ability of HIV-1 to infect hepatocytes (Xiao et al., 2008; Kong et al., 2012; Zerbato et al., 2022). An *in vitro* study verified that HIV-1 can infect hepatic satellite cells (Tuyama et al., 2010). However, no studies yet have shown that cholangiocytes can be infected by HIV-1. Surprisingly, hepatocytes and adipocytes exhibit high HIV-1 ORFs’ CAI. In fact, hepatocytes exhibit the highest overall CAI in all cell types analyzed in this study. On the other hand, hepatic satellite cells which can be infected by HIV-1 *in vitro* exhibit relatively lower HIV-1 ORFs’ CAI. This result seems to indicate that the low susceptibility of adipocytes to HIV-1 is due to the lack of receptors rather than unsuited translational regulatory mechanisms.

#### 3.2.3 Evolutionary analysis of HIV-1 strains based on CAI

**Figure 10:**
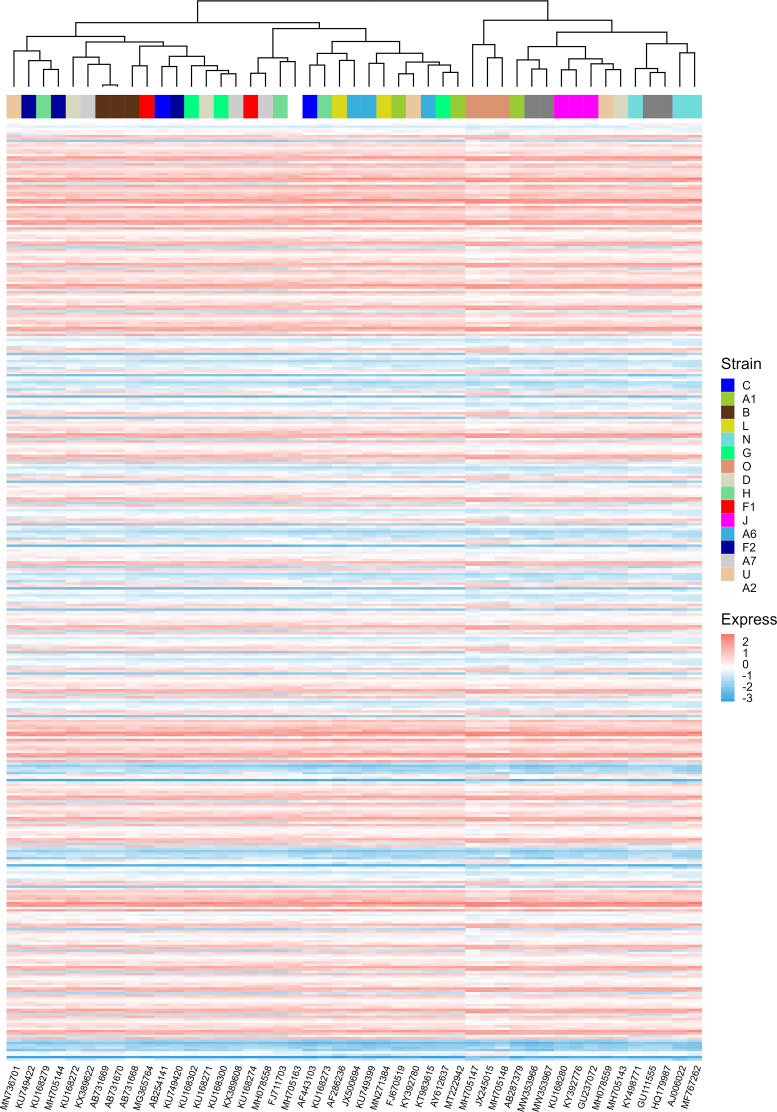
The hierarchical clustering of different HIV-1 strains based on their nine ORFs’ standardized CAIs in all unstimulated cell types/subtypes/large groups from bulk RNA-seq datasets and scRNA-seq datasets. Only strains whose CAIs of all ORFs were calculated were involved in the analysis. Each row represents an ORF in a specific cell type/subtype/large group and each column represents an HIV-1 strain.

During the evolution of viruses, their infectious capacity to different target cells changes gradually. These changes may be manifested in the adaptation of the translational regulatory system of host cells. It was reported that patterns of codon usage can reflect basic features of molecular evolution (Sharp and Matassi, 1994). Therefore, it is possible that ORFs’ CAIs of different HIV-1 strains can reflect the phylogenetic relationship between them. For verifying this hypothesis, we performed a hierarchical clustering analysis of HIV-1 strains based on their ORFs’ standardized CAIs in different cell types (**Figure 10**). However, in the hierarchical clustering analysis, several groups of HIV-1 strains which belong to the same subtype (e.g. L, G, U) are not grouped together. These results indicate that ORFs’ CAIs of HIV-1 strains cannot reflect their phylogenetic history efficiently. This phenomenon may imply that the codon adaptation of HIV-1 ORFs is not selected strongly during the evolution and the adaptation to humans of HIV-1. Thus, it seems efficient translational elongation is not a major limiting factor for HIV-1 infection in humans.

### 3.3 CAI of SARS-CoV-2 and related coronaviruses

#### 3.3.1 The CAIs of different SARS-CoV-2 subtypes

**Figure 11:**
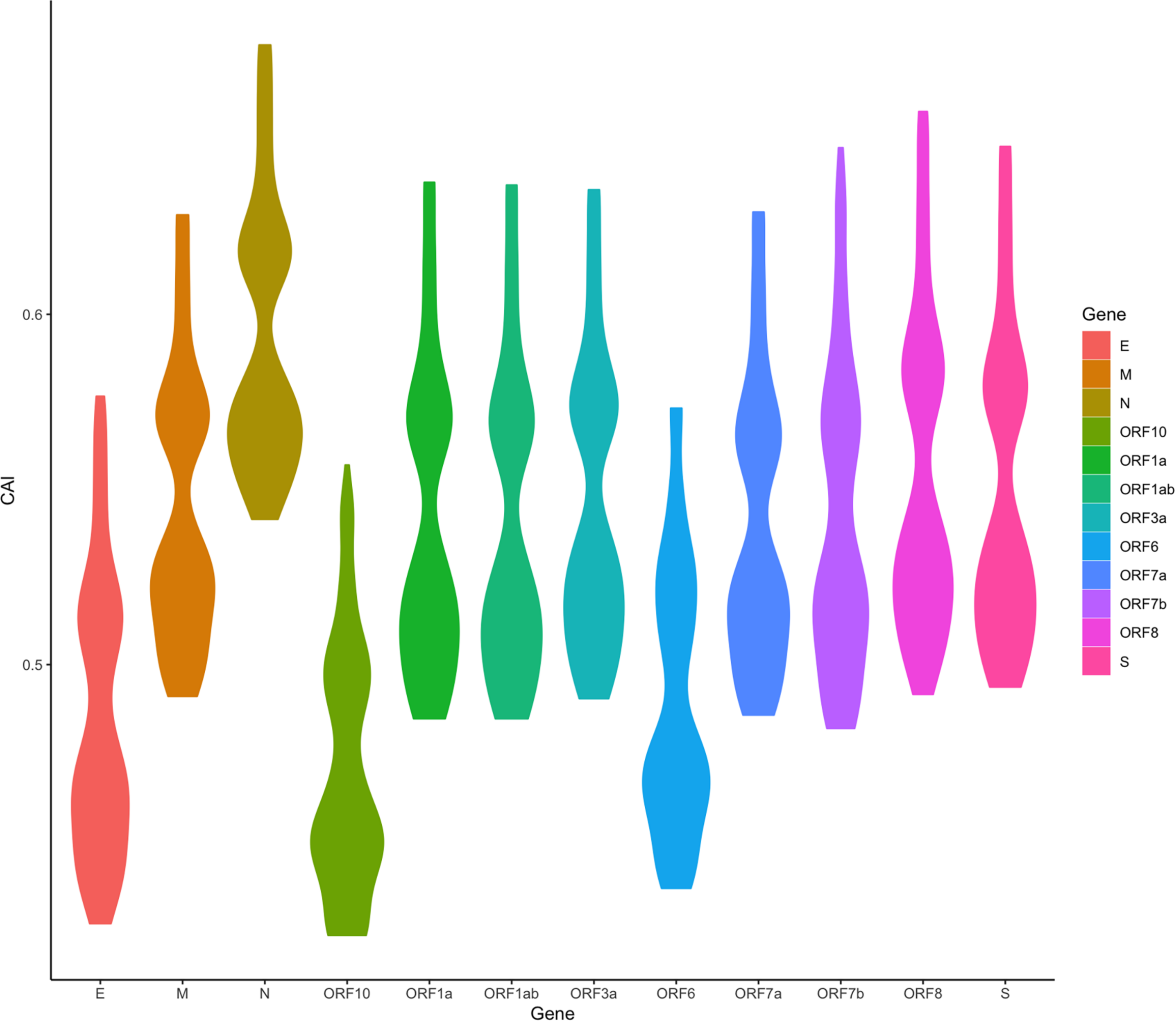
The overall CAI value distribution of different SARS-CoV-2 ORFs in all cell types/large groups from the lung scRNA-seq dataset shown by violin plot. Each violin shows the CAI value distribution of a specific SARS-CoV-2 ORF in all cell types/large groups from the dataset (containing the reference genome and 25 genomes belonging to 5 variants of concern).

We are now focusing on SARS-CoV-2, the pathogen of the COVID-19 pandemic. We calculated the CAI values of SARS-CoV-2 with five VOCs (Alpha, Beta, Gamma, Delta, Omicron) and a reference (original virus identified in Wuhan) in the lung scRNA-seq dataset (Maynard et al., 2020). As discussed earlier, because SARS-CoV-2 infection does not interfere with the relationship between CAI and translational efficiency, this part may reflect both the establishment and the maintenance of SARS-CoV-2 infection. **Figure 11** shows the overall CAI distribution of each ORF of different SARS-CoV-2 subtypes in different lung cells. We can know that, on the whole, the CAI value of the N gene is the highest and the CAI values of E, ORF10 and ORF6 genes are the lowest. However, most of the other ORFs have similar CAIs in the middle. This is basically consistent with the SARS-CoV-2 coding capacity experiment (Finkel et al., 2021), which indicates that the translation efficiency of different ORFs in SARS-CoV-2 is similar. This shows that CAI may be a good indicator of the translation efficiency in SARS-CoV-2. In addition, the overall CAI values of SARS-CoV-2 are lower than most endogenous genes in the previously mentioned three cell types. This result shows the codon adaptation for SARS-CoV-2 in human cells is not sufficient now.

We also found an interesting relationship. Among the essential genes transcribed from subgenome mRNA (sg mRNA), the N gene has the highest expression level, while the E gene has the lowest expression level in the conclusion drawn from the SARS-CoV-2 coding capacity experiment (Finkel et al., 2021). What’s more, the ORF10 and ORF6 genes also show very low expression levels in the coding capacity experiment. Although it is generally believed that CAI does not directly reflect expression levels, these results have an interesting correspondence with CAI results.

**Figure 12:**
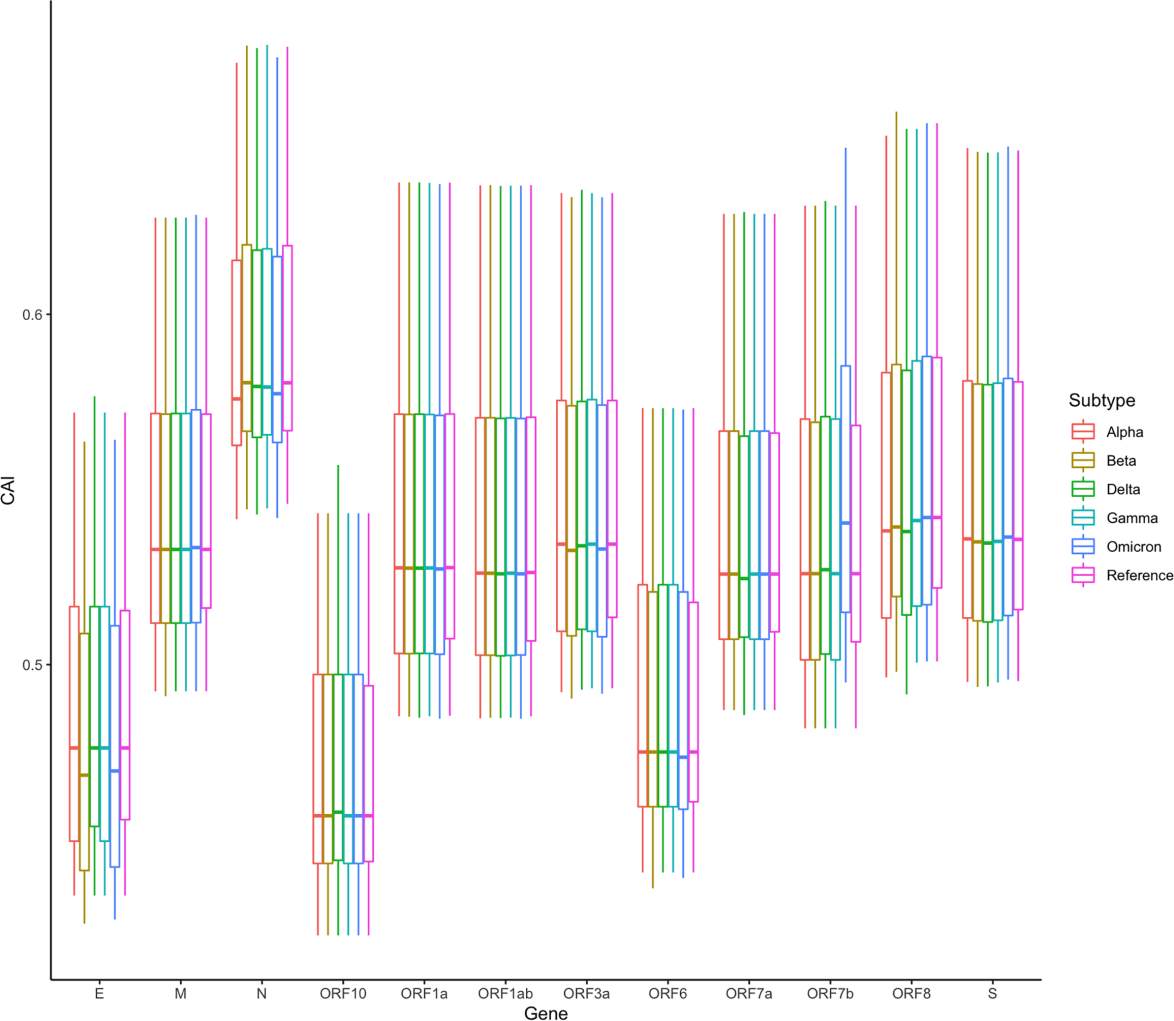
The overall CAI value distribution of SARS-CoV-2 ORFs for different variants of concern (VOCs) in all cell types/large groups from the lung scRNA-seq dataset shown by box plot. Each box shows the CAI value distribution of a specific SARS-CoV-2 ORF for a specific VOC in all cell types/large groups from the dataset.

In addition, we also counted the relative levels of CAI values corresponding to ORFs of different SARS-CoV-2 subtypes in the lung dataset. The results are shown in **Figure 12**. Overall, compared with the reference sequence, the CAI values of other subtypes show a downward trend, which likely indicates that the damage of the virus to the host decreases during the passage. This is consistent with many existing research results (Huang et al., 2021; Barh et al., 2022; McMahan et al., 2022), further proving that CAI can indirectly reflect the damage of the virus. Nevertheless, it should be noted that the mutation of the virus is relatively small now, and its future trend needs further observation. Additionally, in the statistical results, we also find an abnormal situation, that is, the CAI value corresponding to the Omicron ORF7b is significantly higher than that of the reference sequence and other subtypes. The increase in CAI value corresponding to ORF7b may be related to the current pandemics of the Omicron subtype, which needs more experimental research.

**Figure 13:**
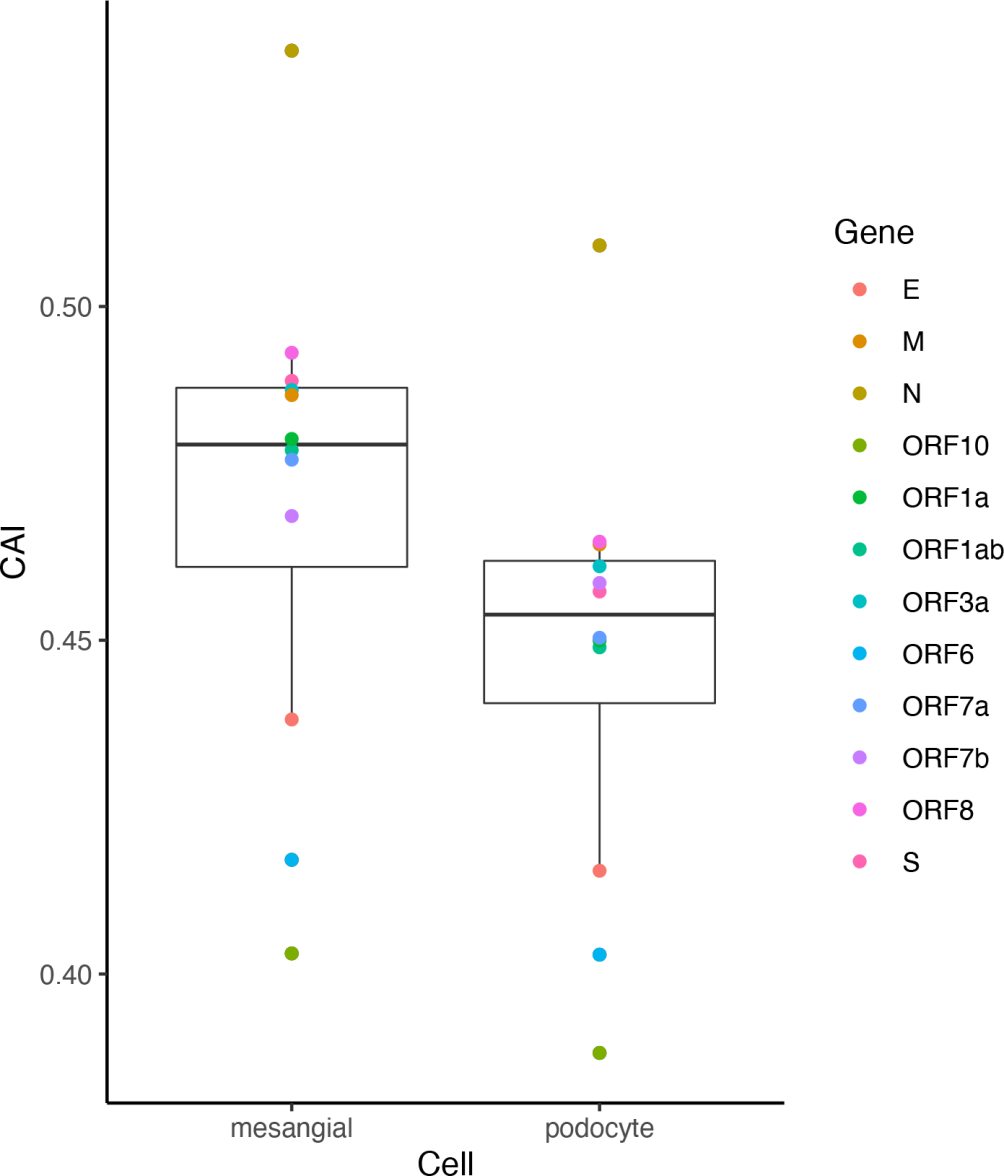
The CAI value distribution of different ORFs of the original SARS-CoV-2 strain in two cell types in the glomerulus (mesangial cells and podocytes) from bulk RNA-seq datasets shown by box plot. Each box shows the CAI value distribution of SARS-CoV-2 reference ORFs in a specific cell type.

Despite significant pulmonary symptoms, COVID-19 is regarded as a systematic disease with symptoms in various organs. For instance, infection of SARS-CoV-2 to the kidney and related acute kidney injury have been reported (Chen et al., 2021). However, renal-related symptoms are less common in COVID-19 than respiratory symptoms. Besides the difficulty of the virus in reaching the kidneys, is there any other explanation for this phenomenon? We analyzed ORFs’ CAI of the original SARS-CoV-2 strain in two cell types from glomerulus (podocytes and mesangial cells; **Figure 13**). Among them, an *in vitro* study has proved that podocytes can be infected by SARS-CoV-2 with the help of ACE2 and BSG/CD147 at their cell surface (Kalejaiye et al., 2022) but no studies have reported that mesangial cells can be infected by SARS-CoV-2. We found that the ORFs of SARS-CoV-2 have very low CAIs in both two types relative to other cell types. Especially, the lowest overall CAIs of SARS-CoV-2 ORFs appear in podocytes among all analyzed cell types. This fact may indicate the difficulty of SARS-CoV-2 to infect these cell types and help to explain why renal-related symptoms are not as common as respiratory symptoms in COVID-19.

#### 3.3.2 The change of SARS-CoV-2 CAIs after viral infection

**Figure 14:**
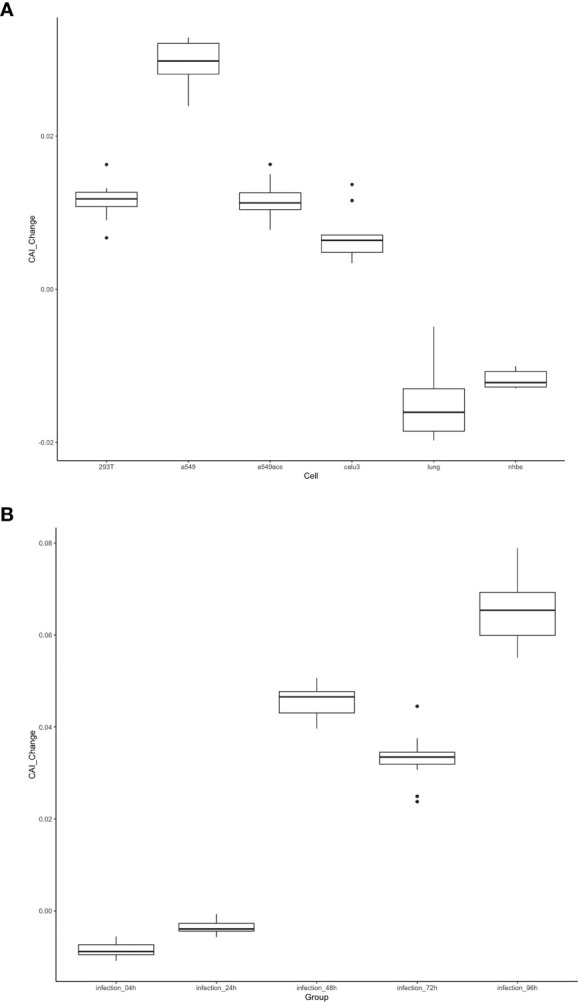
The distribution of the change of CAI values of the SARS-CoV-2 reference genome in different cell datasets shown by box plot. CAI_Change is calculated by CAI in infected cells minus CAI in control cells. **(A)** Each box shows the CAI_Change distribution of all ORFs in a specific cell type (293T, a549, a549ace, calu3, lung, nhbe). **(B)** Each box shows the CAI_Change distribution of all ORFs in infected HBEC cells with different times after infection. The control cells are cultured for 4 hours.

As discussed in the introduction, the inhibition of viral gene translation is a major part of the anti-virus immune response of animal cells (Stern-Ginossar et al., 2019). Therefore, we postulated that the translational efficiency of viral mRNA will be significantly different between uninfected and infected cells. This change may be reflected through CAIs. On the other hand, compared to uninfected cells, CAIs in infected cells can represent the condition in real patients with infectious diseases caused by viruses better. For HIV-1, the correlation between CAI and translational efficiency of endogenous genes is interfered with by viral infection (**Figures 3H and I**), so we did not analyze the change of viral ORFs’ CAIs after HIV-1 infection. However, the correlation between CAI and translational efficiency is maintained in cells infected with SARS-CoV-2. Thus, for studying the effect of viral infection on the translational elongation of SARS-CoV-2 mRNAs, we selected the gene expression datasets of the cells in the control state and the virus-infected state (Daamen et al., 2021; Mulay et al., 2021; Sun et al., 2021; Puray-Chavez et al., 2022), and calculated the CAI values of the SARS-CoV-2 genomes in the corresponding cell datasets. The relative changes of CAI values in the control group and experimental group (virus infection) are consistent in different SARS-CoV-2 subtypes, and the relative positions of CAI values corresponding to ORFs are unchanged. Therefore, using the ORFs of the reference genome for calculation is enough to show the change of cell state after virus infection. It can be seen from **Figure 14** that in more cells, the change of CAI is larger than zero (meaning that the CAI of the experimental group is higher than that of the control group), which indicates that maybe after virus infection, the gene expression of cells changes to be more conducive to virus translational elongation. Additionally, in the dataset of HBEC cells (Puray-Chavez et al., 2022), the CAI values basically increase with the infection time and the CAI values in infected cells are all larger than control cells after 48 hours of infection. This is also strong evidence for the above conclusion.

#### 3.3.3 Comparison of SARS-CoV-2 and other related coronaviruses

Except for SARS-CoV-2, there are six other coronavirus species that can infect humans and cause disease. Among them, HCoV-229E, HCoV-OC43, HCoV-NL63 and HCoV-HKU1 can only infect human cells and just cause mild diseases, while MERS-CoV, SARS-CoV and SARS-CoV-2 are zoonotic in origin and cause severe respiratory illness and fatalities (Hasöksüz et al., 2020). To investigate whether the CAIs of ORFs of these viruses can reflect their difference in pathogenicity and target cells, we calculated the CAIs of their ORFs in cell types from the lung scRNA-seq dataset according to the procedure mentioned earlier.

**Figure 15:**
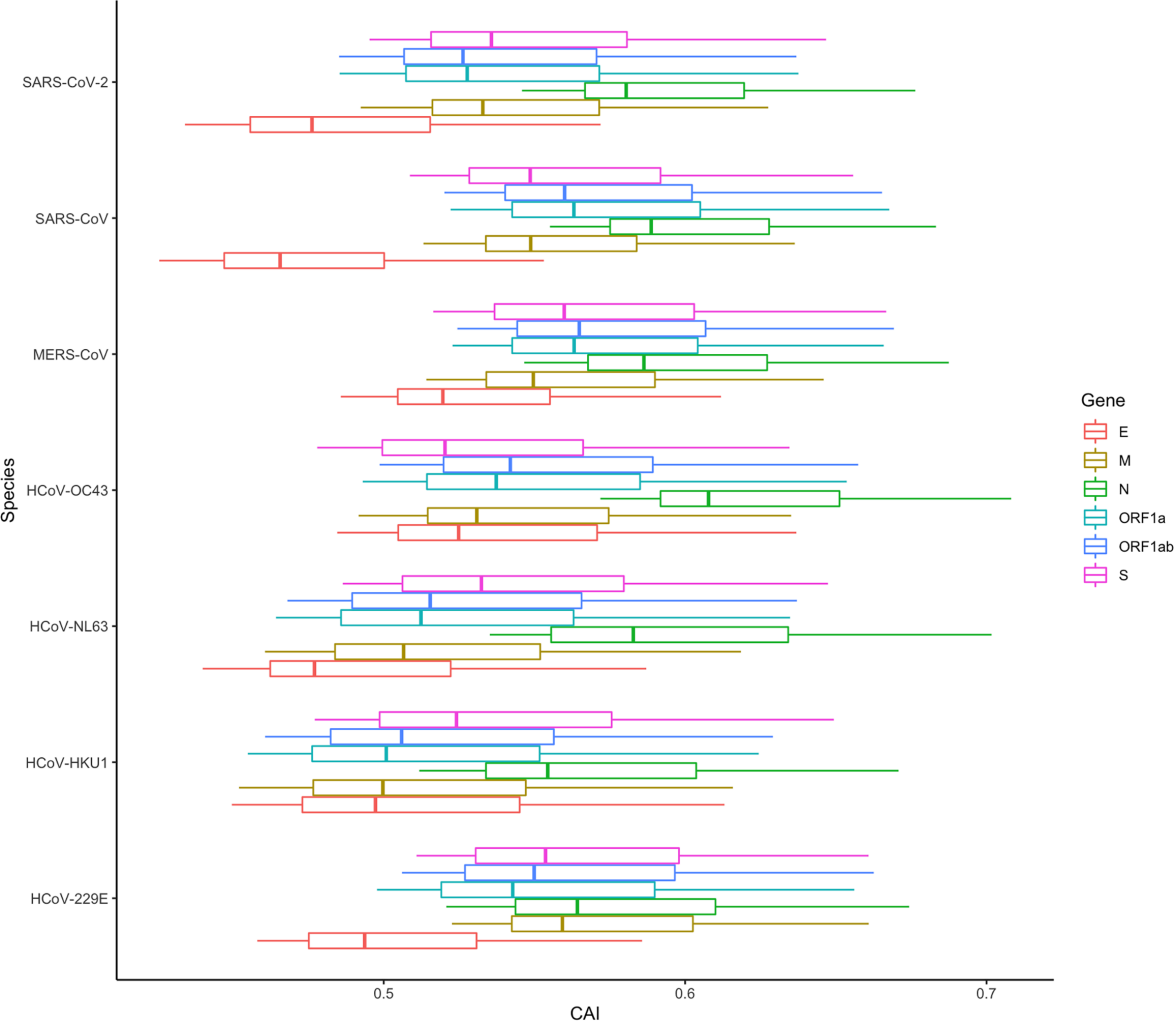
The overall CAI value distribution of ORFs for the reference genomes of seven different coronaviruses in all cell types/large groups from the lung scRNA-seq dataset shown by box plot. Each box shows the CAI value distribution of a specific ORF for a specific coronavirus species in all cell types/large groups from the dataset.

In **Figure 15**, the CAI statistics of 6 essential genes in different cells are shown by boxplots. Similar to SARS-CoV-2, the overall CAI values of these coronaviruses are lower than most endogenous genes in the three cell types mentioned before. This result implies these viruses have not optimized their codon usage for efficient translational elongation in host cells. From **Figure 15**, we can also know that on the whole, the CAI level of MERS-CoV is higher than that of SARS-CoV, and the CAI level of SARS-CoV is higher than that of SARS-CoV-2, which is consistent with the actual pathogenic level of the three (Rabaan et al., 2020). Among the four other human coronaviruses, the overall CAI levels of HCoV-229E and HCoV-OC43 are higher than those of HCoV-NL63 and HCoV-HKU1, which is basically consistent with their actual pathogenic levels (Bouvier et al., 2018). It can be seen that the overall CAI value can reflect the translation level of the virus in the host to a certain extent, and then reflect its pathogenic level. However, we also found that although SARS-CoV-2 can cause pandemics and acute pneumonia, its overall CAI level is slightly lower than HCoV-229E, which means the pandemic and acute pneumonia caused by SARS-CoV-2 may be because of its higher levels of infection and entry ability. But it is also possible that direct comparisons are not appropriate because the viruses infect the host differently (zoonotic/specifically infects humans). In addition, if we compare the relationship among coronaviruses with the same receptor (ACE2: HCoV-NL63, SARS-CoV and SARS-CoV-2; 9-O-acetylsialic acids: HCoV-OC43 and HCoV-HKU1 (Guruprasad, 2021; V'kovski et al., 2021)), the level of CAI value can also perfectly reflect its pathogenic level (overall CAI and pathogenic level: SARS-CoV > SARS-CoV-2 > HCoV-NL63; HCoV-OC43 > HCoV-HKU1).

**Figure 16:**
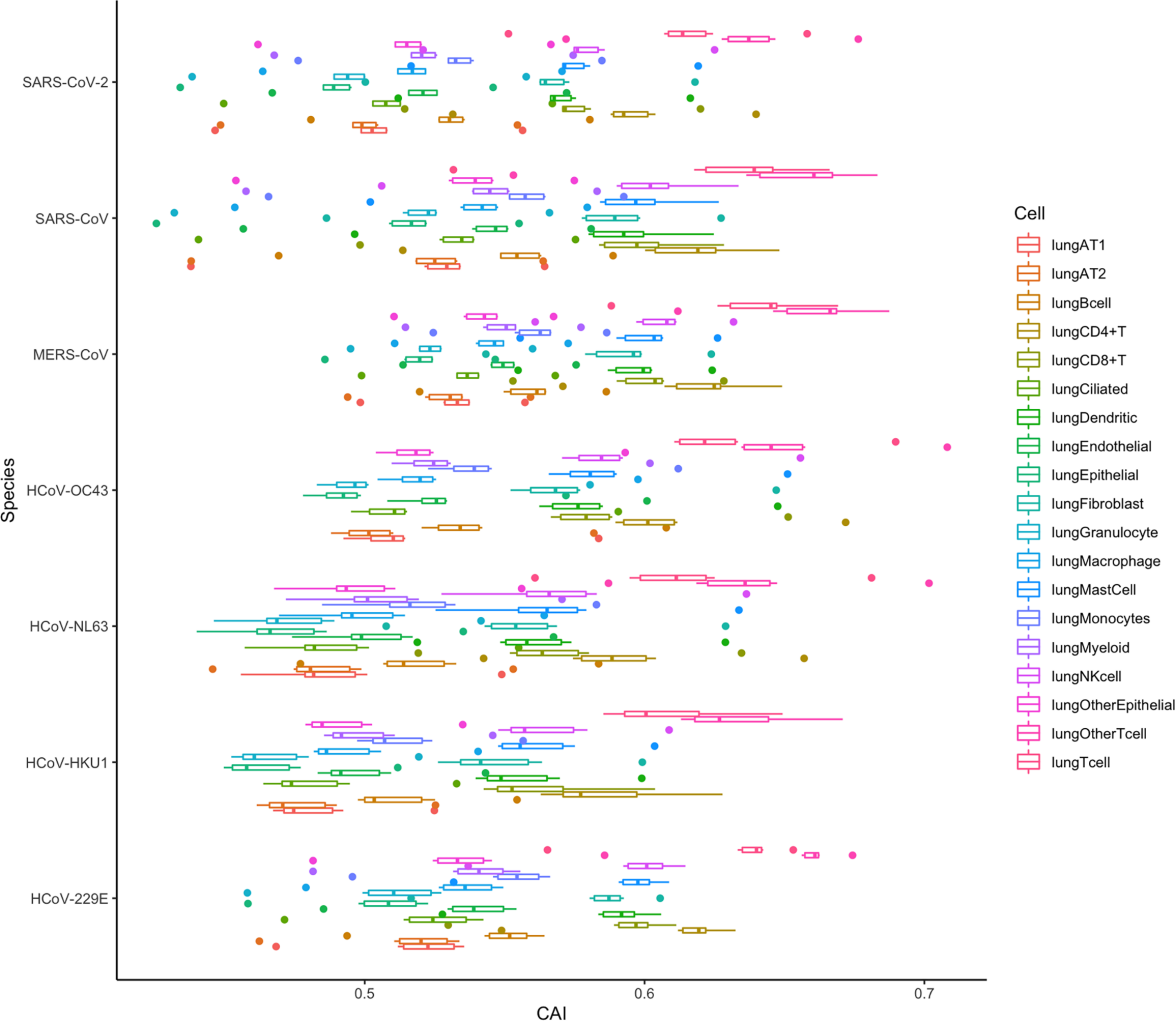
The overall CAI value distribution of all ORFs for the reference genomes of seven different coronaviruses in each cell type/large group from the lung scRNA-seq dataset shown by box plot. Each box shows the overall CAI value distribution of 6 shared essential genes for a specific coronavirus species in a specific cell type/large group from the dataset.

It can be seen from the previous conclusions that in SARS-CoV-2, the CAI is a suitable way to indirectly measure the translation efficiency. Therefore, we tried to verify if the same conclusion was suitable for other coronaviruses by analyzing the CAI distribution of these coronaviruses in different cell types in the lung. The result is shown in **Figure 16** and the data is still derived from 6 shared essential genes. It can be seen that the absolute CAIs of the seven coronaviruses in different cells are somewhat different, but the relative magnitude of the CAI values of each virus for different types of cells is basically stable, which shows the translational adaptation patterns of coronaviruses in human lung cells are likely to be similar.

In addition, we can also find that the CAI values of T cells (lungOtherTcell, lungTcell, lungCD4+T, lungCD8+T), NK cells, and mast cells in lung immune cells are relatively high, suggesting that the destructive power of the coronaviruses inside such cells may be high. This is likely due to the co-evolution of virus and host defense mechanisms during natural evolution. Some existing experimental results can confirm this result, such as SARS-CoV-2, SARS-CoV and MERS-CoV can cause a large decrease in lymphocytes (Gu et al., 2005; Chu et al., 2016; Guan et al., 2020). Infection of host T lymphocytes by SARS-CoV-2 (Shen et al., 2022) and rapid induction of host T lymphocyte apoptosis by MERS-CoV (Chu et al., 2016) have been observed. In SARS-CoV-2 related studies, the damage to host cells is T cell > NK cell > B cell, and for T cell, CD4+ T cell > CD8+ T cell. These results are consistent with the level of their CAI values (Shen et al., 2022). In addition, in the study of MERS-CoV, the virus infection is T cell > NK cell > Monocytes > B cell, which is also consistent with the relative level of CAI values (Chu et al., 2016). Some experiments about SARS-CoV can also support our results, which claim that from their samples, the infected T cells are more than NK cells and B cells (Gu et al., 2005).

However, in the CAI value results, the CAI value of lung AT2 cells is relatively low. And we know that AT2 is one of the main host cells of SARS-CoV-2 and SARS-CoV. This shows that AT2 is their main host cell, probably because the virus can enter such cells more easily. However, its translation efficiency and destruction ability in AT2 cells are probably not higher than those in other cells. In addition, some experiments have shown that T cells are resistant to SARS-CoV infection (Chu et al., 2016). However, in other experiments, SARS-CoV is also found in T cells (Gu et al., 2005). From our results, it is likely that SARS-CoV can adapt to the translation system in T cells better than other cell types. In general, CAI can be used as an auxiliary indicator, together with other indicators, to comprehensively evaluate the ability of the virus to infect and destroy specific cells.

If we evaluate the pathogenic ability of different coronaviruses from the overall CAI value in **Figure 16**, we can draw the conclusion similar to **Figure 15**. This can also indicate that both overall CAI value and CAI for specific ORFs can reflect the translation level of the virus in the host to a certain extent, and then reflect its pathogenic level.

## 4 Discussion and conclusion

In this study, we tried to utilize the CAI to evaluate the infection ability of HIV-1 and SARS-CoV-2 in different cell types. The core hypothesis of this study is if a virus has a higher CAI in a certain type of cell, then it is likely to be better adapted to the translation system of that type of cell. This assumption has been verified by the significantly positive correlation between CAI and translational efficiency of endogenous genes. Compared to the top 5000 highly expressed endogenous genes, the relatively low CAI of viral ORFs indicates that both two viruses have been not well adapted to endogenous translational regulatory machinery in human cells. We also tried to compare the infectious capacity of two kinds of viruses to different human cell types according to CAI. Although many results show that the putative susceptibility to viruses based on CAI is consistent with the results of previous reports, there are still some exceptions. Hence, it seems that at least in the example of HIV-1 and SARS-CoV-2, CAI can provide some clues about cells’ susceptibility to viruses but cannot be used as a single indicator to postulate viral target cells. We also analyzed the effect of viral infection on the cellular translational mechanism and found significant differences between the two viruses. For HIV-1, its infection can decouple CAI and the translational efficiency of endogenous genes. While for SARS-CoV-2, the CAI of its ORFs increases after the establishment of infection. It is expected that both mechanisms are beneficial for the virus to overcome the defects in codon adaptation in human cells and achieve effective gene expression.

Compared with previous studies that evaluated the infectivity of viruses based on codon adaptation, the main advantage of our study is that our analysis is refined to the cell-type level. First, previous studies usually selected a predefined set of highly expressed genes (usually housekeeping genes, such as ribosomal genes) (Ruiz et al., 2006; Tello et al., 2013; Khandia et al., 2019) as the background gene set to assess the susceptibility of a species to viruses. However, for multicellular organisms such as humans, the gene expression patterns of different cell types and their regulatory mechanisms, including translation extension, are significantly different, so it would be unreasonable to use the same set of background genes. A few studies (Mogro et al., 2022) went deeper into the organ level and selected the corresponding background gene sets according to the gene expression patterns of different organs in an attempt to analyze the susceptibility of different organs to viruses based on this. However, animal organs are not uniform and compose of a large number of cells with different functions and gene expression patterns. Different types of cells in the same organ have different susceptibilities to viruses. For example, alveolar type II epithelial cells with a high expression level of ACE2 are the main target cells of SARS-CoV-2 in the lung. Therefore, an analysis based on isolated specific cell types may provide a more reasonable assessment of viral infection patterns *in vivo* than the analysis based on whole organs. In our study, we quantitatively analyzed the gene expression levels of different cell types in the human body based on bulk RNA-seq and single-cell RNA-seq data and obtained the specific high-expression gene sets to each cell type for CAI analysis. Thus, compared with previous similar studies, our study may reveal the translational elongation activity of the viral ORFs in different human cell types and estimate the susceptibility of these cell types to viruses.

There are several reasons which may explain why codon adaptation cannot be used as a single indicator to postulate viral target cells. First, as we discussed in the ‘Results’ part, the translational efficiency may not be the limiting factor for these two viruses to infect human cells. For example, in our analysis of the scRNA-seq dataset of PBMC, HIV-1 ORFs exhibit the highest CAI in B cell which is not susceptible to HIV-1. This contradiction can be simply attributed to the lack of CD4 or other possible HIV-1 receptors at B cell surfaces. Second, regulation of gene expression is due to complex factors in multilevel. It is possible that differences in regulatory mechanisms of gene expression in different human cell types are mainly manifested in pre-translation (epigenomic or transcriptional) and translation initiation levels. However, CAI mainly reflects the regulation at the translational elongation (codon adaptation) level so it cannot summarize all regulatory mechanisms which can affect the expression of ORFs in the virus genome. Third, viruses can manipulate the translation machinery of cells e.g., through modulating cellular tRNA pools (Anna et al., 2011). This process can improve the translational efficiency of viral ORFs and decouple the relationship between CAI and translational efficiency. Finally, as shown in **Figure 3**, in different human cell types, the relationship between CAI and translational efficiency of endogenous genes is significantly different, thus the comparability of CAI among different cell types needs further study.

In conclusion, we calculated CAI for two kinds of pandemic RNA viruses in the background of different human cell types. Although previous studies related to CAI or other indexes utilized in the translational efficiency estimation mostly stayed at the species level, our studies refined the estimation of translation efficiency by CAI calculation to the cell type level. We found that although CAI cannot be used as the only indicator to postulate viral target cells alone, it really provides some clues about cells’ susceptibility to viruses from the aspect of codon adaptation. Additionally, we took CAI calculations down to the cellular level for HIV-1 and SARS-CoV-2, and considered alternative splicing in the construction of high-expression gene sets in each cell type. This is beneficial to make the calculation of CAI more reasonable. Next, we presented the CAI patterns of these two viruses in various cells. In addition, we also studied the changes of CAI before and after viral infection, and found that in most datasets, the gene expression of cells after viral infection will lead to the increase of SARS-CoV-2 ORFs' CAIs, which indicates that the translational system may be more suitable for the virus translational elongation after viral infection. Furthermore, both viruses were found to have the highest CAI values in immune cells (T cells), suggesting a co-evolutionary or adaptive relationship between viruses and immune cells.

## Data availability statement

The datasets presented in this study can be found in online repositories. The names of the repository/repositories and accession number(s) can be found in the article/**Supplementary Material**.

## Author contributions

HZ (first author): programming, data curation, result analysis and visualization, writing - original draft, and revising. RR (first author): programming, result analysis and visualization, writing - original draft, and revising. SY (corresponding author): conceptualization, methodology, funding acquisition, project administration, writing - review & editing. All authors contributed to the article and approved the submitted version.
