# Supplementary material for: Utilizing the codon adaptation index to evaluate the susceptibility to HIV-1 and SARS-CoV-2 related coronaviruses in possible target cells in humans
Source: Front Cell Infect Microbiol. 2023 Jan 25;12:1085397. doi: 10.3389/fcimb.2022.1085397 (PMC9905242; doi:10.3389/fcimb.2022.1085397)
Supplement: Supplementary file 1 [file DataSheet_1.pdf]

Methods

1. Calculation of the codon adaptation index (CAI)

For a specific RNA in a specific cell type with corresponding high-expression protein-coding (Unless otherwise stated, the term 'gene' below only refers to protein-coding genes) geneset, the expression of the original version of CAI while it was proposed [21] is:

$$CAI = \left(\prod_{k=1}^{L} \frac{p_k}{q_k}\right)^{\frac{1}{L}}$$

'L' is the number of sense codons (except ATG, the only codon for Met, and TGG, the only codon for Trp) in the ORF of concerned RNA. 'p_k' is the usage frequency of the k-th sense codon (from the initiation codon to the last sense codon before the termination codon) in the ORF of concerned RNA in all synonym codons of the high-expression geneset. 'q_k' is the max usage frequency of the synonym codon of the k-th codon in the ORF of concerned RNA in all synonym codons of the high-expression geneset.

The original version of CAI was used widely to evaluate the translational efficiency of mRNA. However, it has two major problems [25]. First, if the usage frequency of a specific codon from the concerned RNA in the high-expression geneset is zero, the CAI of this RNA will be zero regardless of the composition of its other codons [25]. Then the function of CAI in evaluating translation efficiency will be lost. For avoiding this condition, we can convert p_k and q_k in the CAI expression to $p_k+0.01$ and $q_k+0.01$. Second, for three kinds of amino acids with six corresponding codons (Ser, Arg and Leu), their codons can be divided into two smaller groups according to the first two bases. In these conditions, the same amino acid coded by different groups of codons can be considered as different amino acids in the calculation of CAI [25]. For the CAI calculation in our study, both two improvements mentioned above were adopted.

2. Constructing the high-expression geneset of each concerned cell type

2.1 Collection of the human RNA-seq data of concerned cell types.

For constructing high-expression genesets, measuring the expression level of genes in different cell types is necessary. Transcriptomic techniques, e.g. bulk and single-cell RNA-seq, are the main methods to measure gene expression levels in tissues or cells currently [26]. In our study, human RNA-seq datasets of different cell types (shown in Table 1) from the NCBI GEO database (https://www.ncbi.nlm.nih.gov/geo/) were selected for constructing corresponding datasets.

Table 1

In order to make the subsequent analysis feasible and reasonable, we have designed several criteria for selecting RNA-seq datasets: (1) For ensuring the representativeness of data, the number of biological repetitions in bulk RNA-seq datasets should be greater than or equal to three. (2) Because of the need of discriminating different alternative splicing mRNA isoforms for each gene, for bulk RNA-seq datasets, only datasets that utilized poly-T oligomers to capture mature spliced mRNAs or exhibited similar successfully assigned alignments ratio while quantitating RNA transcripts with exon or gene features (This means sequencing reads which align to intron or immature RNA will not affect the results of alternative splicing analysis significantly) in featurecounts (see below) are selected for analysis. For single-cell RNA-seq datasets, only datasets that utilized the Smart-seq2 sequencing protocol which can sequence the overall length of mRNA are selected for analysis. (3) For the same reason in (2), only datasets with accessible raw sequencing data (sra format in the NCBI SRA database https://www.ncbi.nlm.nih.gov/sra) which can be utilized in RNA alternative splicing analysis are selected. It should be noted that according to medical ethics, for respecting the privacy of patients, raw data of lots of human datasets are not provided. (4) For ensuring that gene expression patterns of samples analyzed are consistent with *in vivo* conditions, except for three datasets which include data from *in vitro* differentiated macrophages, dendritic cells and osteoclasts (seen in Table 1), samples of all unstimulated datasets are cells directly from patients or primary cultured cells. This criterion is not suitable for cytokine-stimulated or viral-infected datasets because *in vivo* stimulation of human cells by viruses or cytokines is unethical. Finally, 19 bulk RNA-seq datasets (including 2 datasets with corresponding Ribo-seq data) corresponding to different types of cells and 2 single-cell RNA-seq datasets corresponding to major target ‘organs’ of SARS-CoV-2 and HIV-1 (lung and PBMC respectively) were selected.

2.2 Gene-level expression analysis of RNA-seq datasets

To determine gene expression levels by RNA-seq, a series of processes are needed to convert raw sequencing data into a matrix of gene expression. The pipeline of

RNA-seq data analysis in our study is mentioned below: fastq-dump in NCBI SRA toolkit (version: 2.11.3) was utilized to split sra files into fastq files and fastqc (version: 0.11.9) was used to evaluate the quality of sequencing data (Because the number of human RNA-seq datasets with accessible raw data is relative low, the quality evaluation results from fastqc were not used as a criterion for selecting datasets). For RNA-seq datasets, hisat2 [41] (version: 2.2.1) was utilized to align sequencing reads in fastq files to the human reference genome (version: hg38) and produce sam files. Sam files outputted by hisat2 were compressed into bam files by samtools [42] (version: 1.15). Then featurecounts [43] in subread (version: 2.0.3) was utilized to quantify gene expression levels from bam files according to the gene-level genome annotation file (version: 39) and outputted the gene expression (counts) matrix. Finally, the expression level of each gene by counts in each sample (for bulk RNA-seq. For each biological repetition, the gene expression level of all technical repetitions are added together to generate a single sample.) or cell (for single-cell RNA-seq) was converted to normalized values (FPKM) in R according to the accumulated exon length from the genome annotation file. For single-cell RNA-seq data, the R package Seurat (version: 4.1.1) was used to preprocess, cluster and visualize cells according to their gene expression patterns. Cell types were annotated according to the corresponding literature of the dataset. Only cell types with more than ten cells in each single-cell RNA-seq dataset were used for CAI calculation and further analysis. Gene-level expression data (raw counts and FPKM) for all RNA-seq datasets used in this study is provided in Github. For two single-cell RNA-seq datasets, the corresponding relationship between cells and cell types/large groups was also listed in Github. Also, intermediate results of single-cell RNA-seq datasets by Seurat including cell clustering and visualization in low-dimension space, ratio or number of cells belonging to each cell type/large group and markers to identify each cell type/large group were shown in Supplemental Fig. 1 and Fig. 2.

2.3 Select high-expression genes in each concerned cell type

According to the concept of CAI, the definition of the high-expression geneset is arbitrary. In previous studies, typical housekeeping genes like ribosomal genes and histone genes are usually utilized to construct the high-expression geneset. The lack of cell type-specificity made this strategy unsuitable for our study. For highlighting differences in gene expression patterns in various cell types, we selected the top 200 protein-coding genes (contain both housekeeping genes and genes with tissue/cell-specific expression) with the highest mean FPKM level in biological repetitions (for bulk RNA-seq datasets) or cells (for single-cell RNA-seq datasets) for a cell type to construct the high-expression geneset of this cell type. Also, we found that expression levels of some genes changed significantly in different biological repetitions or cells of the same cell type. Hence, before selecting high-expression genes as mentioned above, we removed genes whose standard deviations of the expression levels are large than the average value. The selected top 200

high-expression genesets and their normalized expression level (FPKM) for all RNA-seq datasets used in this study are provided in Supplemental File 1. To characterize the functional characteristics of high-expression genesets in different cell types, we performed GO-BP and KEGG enrichment analysis on them by R package clusterProfiler [44] (version: 4.0.5) and Org.Hs.eg.db (version: 3.13.0). Results of enrichment analysis for three representative datasets (bloodmonocytes, CD4+ Tfh in bulk RNA-seq datasets and dendritic cells in scRNA-seq datasets) are provided in Supplemental File 2.

2.4 Alternative splicing analysis of the high-expression genes

Different from prokaryotic genes, mRNAs transcripted from eukaryotic genes usually experience complex processing including splicing. Because of the existence of alternative splicing, the same mRNA precursor can be processed to different mature mRNAs and translated to different protein products. As a result, it is necessary to discriminate different splicing isoforms of genes in the high-expression geneset. For this purpose, we utilized featurecounts to quantify exon expression levels from previously mentioned bam files according to the exon-level genome annotation file (modified by the previous gene-level genome annotation file through methods mentioned in https://github.com/vivekbhr/Subread_to_DEXSeq) and output the exon expression (counts) matrix. For each spliced isoform with annotation in NCBI or UNIPROT, its approximate expression counts were calculated by adding counts of all exons that are parts of this isoform together. Then approximate counts of each isoform were converted to approximate FPKM according to the length of each isoform from the exon-level genome annotation file. For each gene, only the isoform with the highest approximate FPKM of each gene was utilized to construct the codon usage frequency table of the high-expression geneset, then p_k and q_k in this expression of CAI could be acquired from this table. CDS sequences of the isoform with the highest approximate FPKM of each gene in high-expression genesets for all RNA-seq datasets used in this study are provided in Supplemental File 3.

2.5 Verifying the relationship between CAI and translational efficiency of endogenous genes

For verifying if CAI can represent genes' translational efficiency, we calculated the CAI of the top 5000 highly expressed genes (except genes that are not detected in Ribo-seq experiments) in two groups of paired RNA-seq and Ribo-seq datasets of control cells and cells infected with HIV-1 or SARS-CoV-2 for a certain time. Also, we calculated the translational efficiency by dividing their normalized expression values (FPKM) in Ribo-seq datasets by values in RNA-seq datasets of these genes

and then took the logarithm. Then we performed a linear regression analysis between genes' CAI and translational efficiency in each cell type. Major data processing steps including gene-level expression and alternative splicing analysis for Ribo-seq datasets are similar to RNA-seq datasets mentioned in 2.3. The only difference is that STAR [45] (version: 2.7.9) instead of hisat2 was used to perform sequencing reads alignment and produce sam files because it is more sensitive to short reads produced by Ribo-seq. Furthermore, for verifying if CAI of endogenous genes calculated by celltype-specific background genesets correlated better with translational efficiency than CAI based on commonly used nonspecific background genesets, we constructed a nonspecific background geneset reference to previous mentioned methods. First, typical housekeeping genes-ribosomal protein genes are selected. Then, we filtered them according to expression patterns, preserving only the ribosomal genes that were present in the top 200 highly expressed genes of at least one cell type (except cells infected by virus or stimulated by cytokines and paired control groups in the same dataset) . The CAI of endogenous genes based on this nonspecific background geneset and the correlation between this 'nonspecific' CAI and translational efficiency is calculated in the same way as above. In CAI calculation, for each gene in the non-specific background geneset, one isoform from all isoforms appear in the top 200 highly expressed geneset of at least one cell type was selected randomly and utilized in the construction of the the codon usage frequency table.

Gene-level expression data (raw counts and FPKM) for all Ribo-seq datasets used in this study is provided in Github page. The CAIs and the translational efficiency of the top 5000 highly-expressed genes for all RNA-seq/Ribo-seq pair datasets in this study are provided in Supplemental File 4.

3. Collecting genomic and ORF sequences of HIV-1, SARS-CoV-2 and other coronaviruses

For HIV-1, genomic sequences were collected from the HIV sequence database [46] (https://www.hiv.lanl. gov/content/sequence/HIV/mainpage.html). There are 21 known subtypes (A1, A2, A3, A4, A6, A7, A8, B, C, D, F1, F2, G, H, J, K, L, N, O, P, U) for HIV-1. Because all genome sequences of subtype K in the database do not meet our standards, this subtype of viruses was not collected and included in the analysis below. For every other subtype, 2-3 genomic sequences were collected to remove random bias. Finally, 58 genomic sequences of HIV-1 were collected for further analysis.

There are three ORFs for essential primary protein products (gag, gag-pol, env. Among them, the front part of gag-pol ORF is the same as gag and the latter part differs from gag by a frame-shift mechanism) and six ORFs for non-essential primary

protein products (vif, vpr, tat, rev, vpu, nef) in HIV genomes. However, not all HIV-1 genomes in the database contain all nine functional ORFs. Just as its name implies, the absence of non-essential primary protein products will not prevent the virus from replicating and proliferating. Also, some non-functional viral genomes with a serious deficiency in essential ORFs are collected by the database. On the other hand, because of technical problems during sequencing, there are deletions at the end of some genome sequences collected. Thus, the 5-terminal part of gag/gag-pol ORF or the 3-terminal part of nef ORF in some of these sequences is incomplete. Considering the above situations, we design a series of standards to exclude some 'problematic' ORFs in CAI calculation and further analysis:

(1) In the condition in which the 5-terminal (for gag/gag-pol) part or 3-terminal (for nef) part of the ORF is incomplete, if the length of deleted part exceeds 5% of the ORF's total length, this ORF will be excluded.
(2) In the condition in which the ORF is annotated to be nonfunctional due to the mutation, if the mutation is nonsense (create a termination codon that causes the early termination of translation), frameshift or the initiation codon is removed due to the mutation, this ORF will be excluded. Conversely, if the mutation is missense, this ORF will be retained.
(3) In the condition in which the ORF (exactly, genomic region) is annotated as 'similar to a gene', the standard of excluding ORF is similar to (2). If the region's length is significantly different from functional ORF (usually due to nonsense mutation) or the initiation/termination codon is lacking, the ORF will be excluded.

Major information (patient ID, accession ID, name, subtype, country and year for separation, completeness and length for genomic sequence and annotation for specific conditions) and ORFs' sequences for all 58 HIV-1 strains used in this study are provided in Supplemental File 5. If one ORF is regarded as 'problematic' according to the above standards and excluded in CAI calculation, this ORF will be marked as "nonfunction" in the table.

For SARS-CoV-2, the genomic sequence of the original strain in Wuhan was collected from the NCBI nucleotide database (https: //www.ncbi.nlm.nih.gov/nuccore) and five variants of concerns (Alpha, Beta, Gamma, Delta and Omicron) identified by the World Health Organization (WHO) were collected from GISAID influenza virus and SARS-CoV-2 sequences database (https://www.gisaid.org). For each variant of concern, 5 genomic sequences were collected to remove random bias. There are six ORFs for essential primary protein products (ORF1a, ORF1ab, S, E, M, N. The relationship between ORF1ab and ORF1a is similar to the relationship between gag-pol and gag in the example of HIV-1) and six ORFs for non-essential primary protein products (ORF3a, ORF6, ORF7a, ORF7b, ORF8, ORF10) in SARS-CoV-2 genomes [47]. Although the deficiency of non-essential ORFs is common in genomes of some SARS-CoV-2 strains, all ORFs are complete and functional in SARS-CoV-2 genomic sequences collected by our studies. Therefore, all twelve ORFs of these

sequences are involved in downstream analysis. Major information (accession ID, name, VOCs, country and time for separation) and ORFs' sequences for all 26 SARS-CoV-2 strains used in this study are provided in Supplemental File 6.

For six other coronavirus species (SARS-CoV, MERS-CoV, HCoV-OC43, HCoV-NL63, HCoV-HKU1, HCoV-229E) that can infect humans, genomic sequences were collected from the NCBI nucleotide database. For each species, only the reference genomic sequence was collected. For each of these sequences, all ORFs annotated in corresponding species are functional and are involved in downstream analysis. ORFs' sequences for all six other coronaviruses used in this study are provided in Supplemental File 7.

4. Calculation and Downstream analysis of viral ORFs' CAI

After sequence collection, the CAI of ORFs of all HIV-1, SARS-CoV-2 and other coronavirus strains above was calculated according to Part 1 in Methods. Results for three groups of viruses are provided in Supplemental File 8-10 separately. Relative usage frequency of synonymous codons (relative synonymous codon usage, RSCU) of top 200 high-expressed genes in all cell types which is needed for CAI calculation is in Supplemental File 11.

For HIV-1, we compared the CAIs of their ORFs in several groups of different cell types (1) 16 cell types and 3 large groups in the lung. (2) 7 cell types of peripheral blood mononuclear cells (PBMC) (3) cell types of the monocyte-macrophage system (4) 4 subtypes of CD4+ T lymphocytes (5) 5 cell types from two key metabolic organs. To test if the CAI can reflect the evolutionary relationship of different viral strains, hierarchical clustering algorithms were utilized to cluster HIV-1 viral strains' ORFs based on their ORFs' standardized (z-score) CAIs in different cell types. For visualizing the results of our analysis, the R package ggplot2 (version: 3.3.6) was utilized.

For SARS-CoV-2 subtypes and six other coronavirus species, we compared the CAIs of their ORFs in the lung dataset (16 cell types and 3 large groups in the lung). We also showed the CAI distribution of SARS-CoV-2 in glomerulus-podocytes and mesangial cells because kidney were reported as an affected organ of COVID-19 [11]. In addition, we hoped to explore the change of CAI to observe the change of cell state after viral infection. Therefore, we also calculated the CAIs of SARS-CoV-2 ORFs in the Bulk RNA-seq datasets of infected and control human cells. For visualizing the results of our analysis, the R package ggplot2 (version: 3.3.6) was utilized.
