# Supplementary figures and images for: Utilizing the codon adaptation index to evaluate the susceptibility to HIV-1 and SARS-CoV-2 related coronaviruses in possible target cells in humans
Source: Front Cell Infect Microbiol. 2023 Jan 25;12:1085397. doi: 10.3389/fcimb.2022.1085397 (PMC9905242; doi:10.3389/fcimb.2022.1085397)

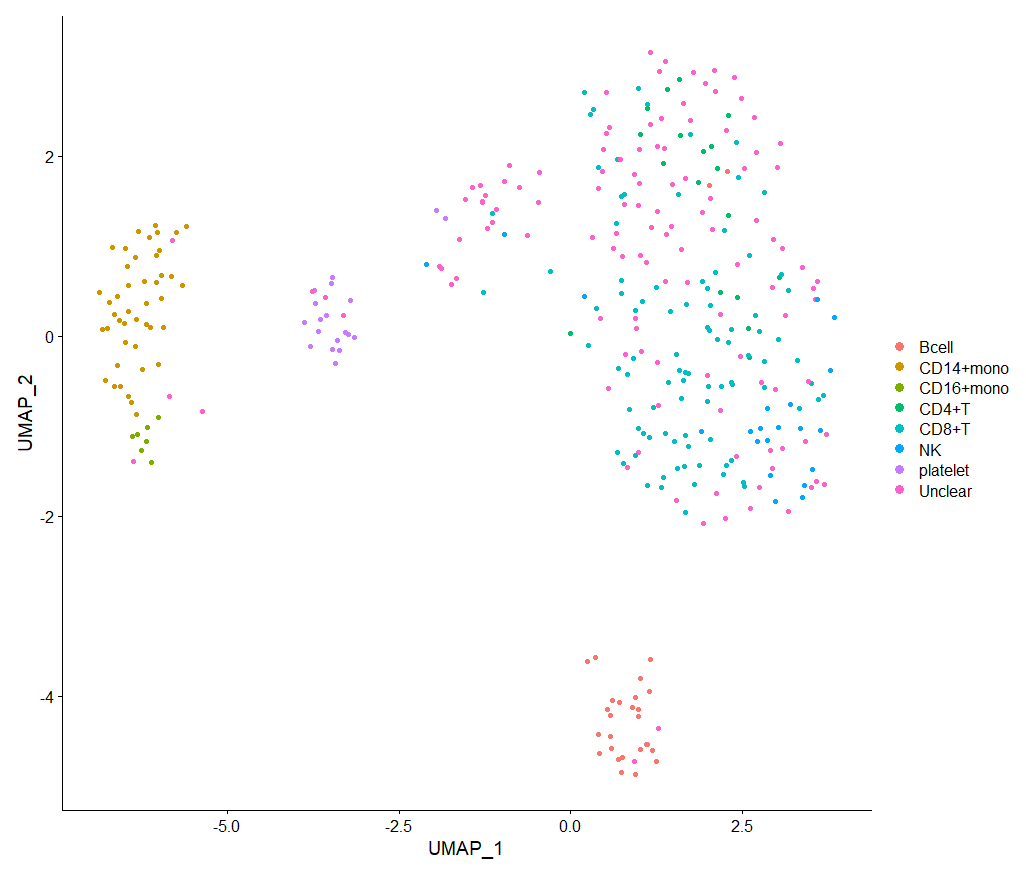

Supplement: Supplementary file 2 [file DataSheet_2.zip › Supplemental Figures/Fig S2/Fig 2A.png]

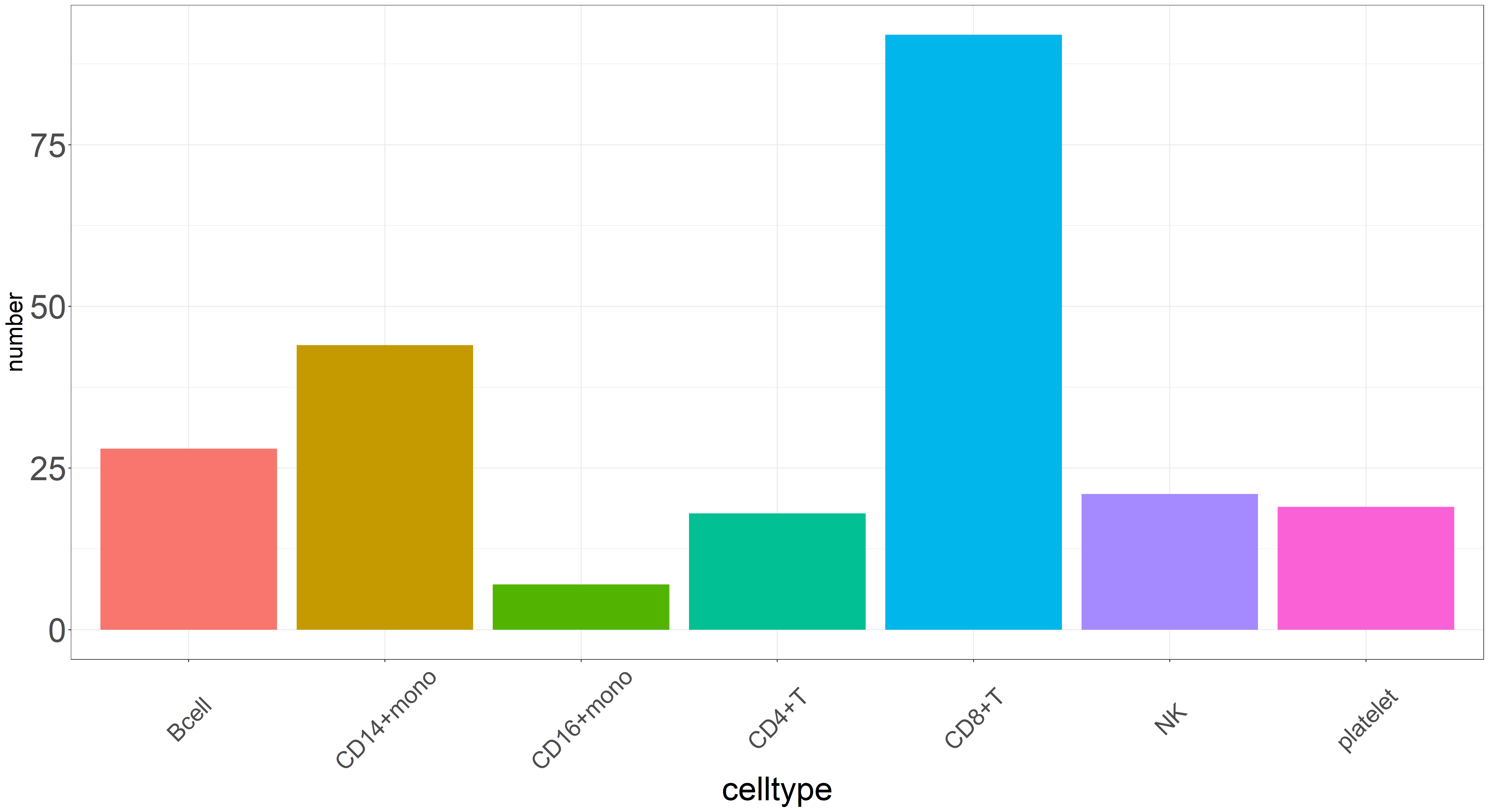

Supplement: Supplementary file 2 [file DataSheet_2.zip › Supplemental Figures/Fig S2/Fig 2C.png]

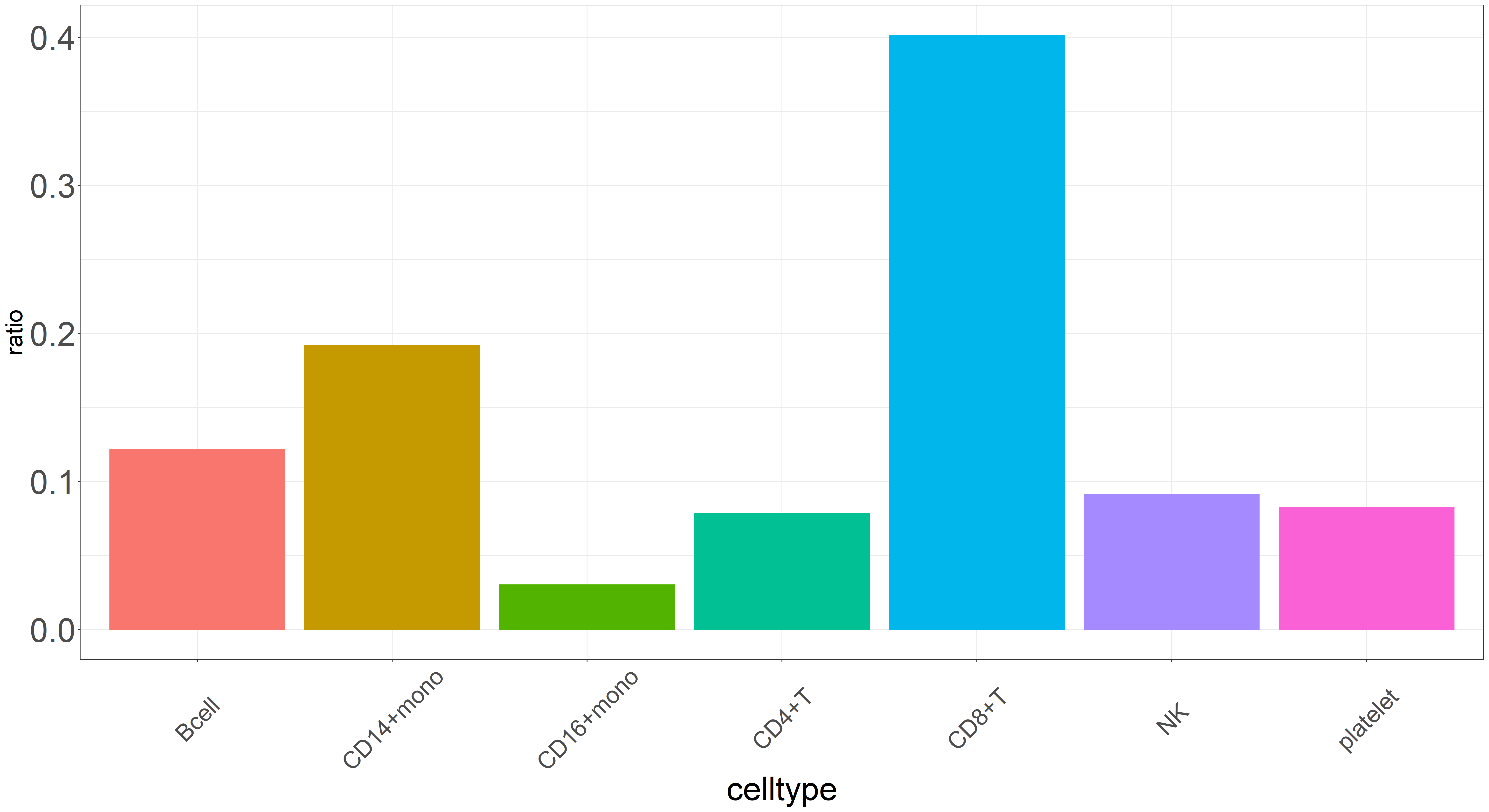

Supplement: Supplementary file 2 [file DataSheet_2.zip › Supplemental Figures/Fig S2/Fig 2D.png]

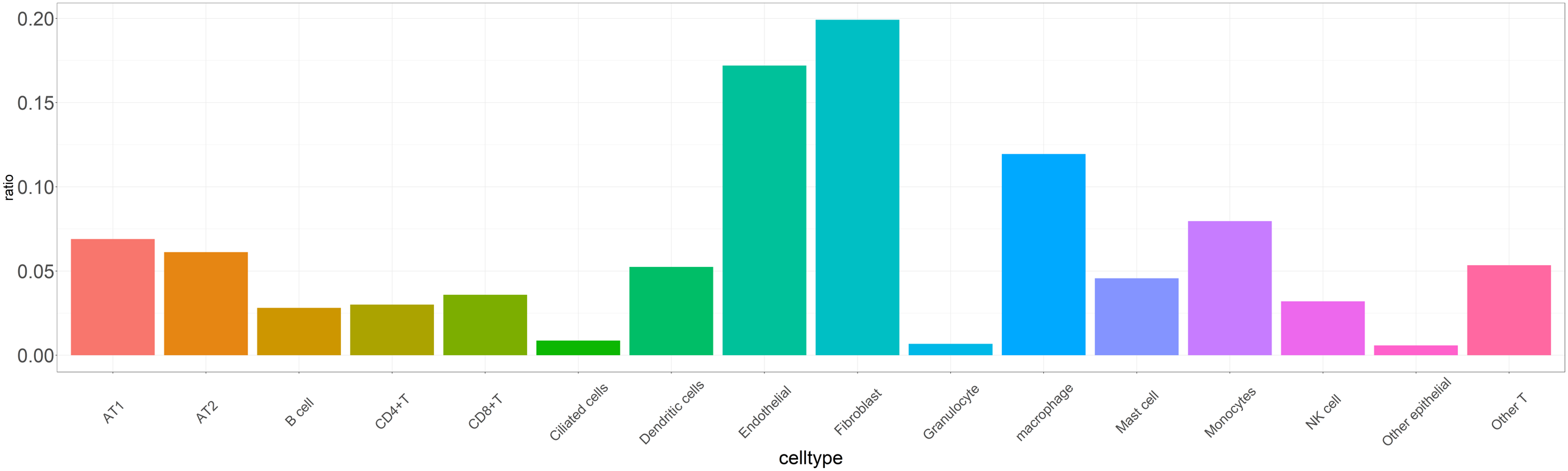

Supplement: Supplementary file 2 [file DataSheet_2.zip › Supplemental Figures/Fig S1/Fig 1E.png]

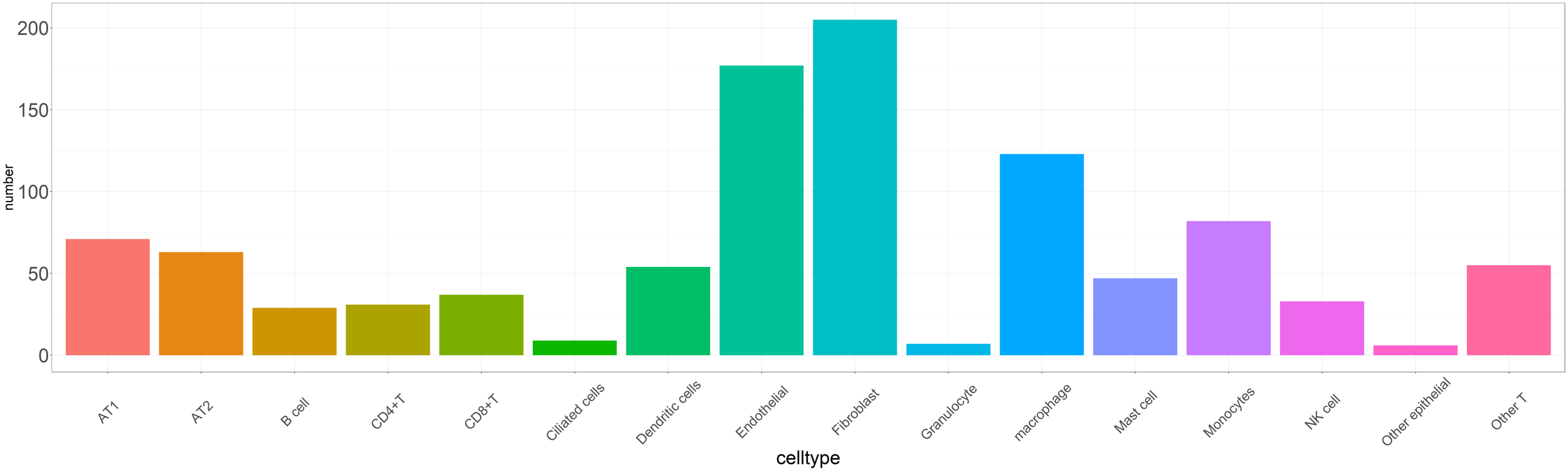

Supplement: Supplementary file 2 [file DataSheet_2.zip › Supplemental Figures/Fig S1/Fig 1D.png]

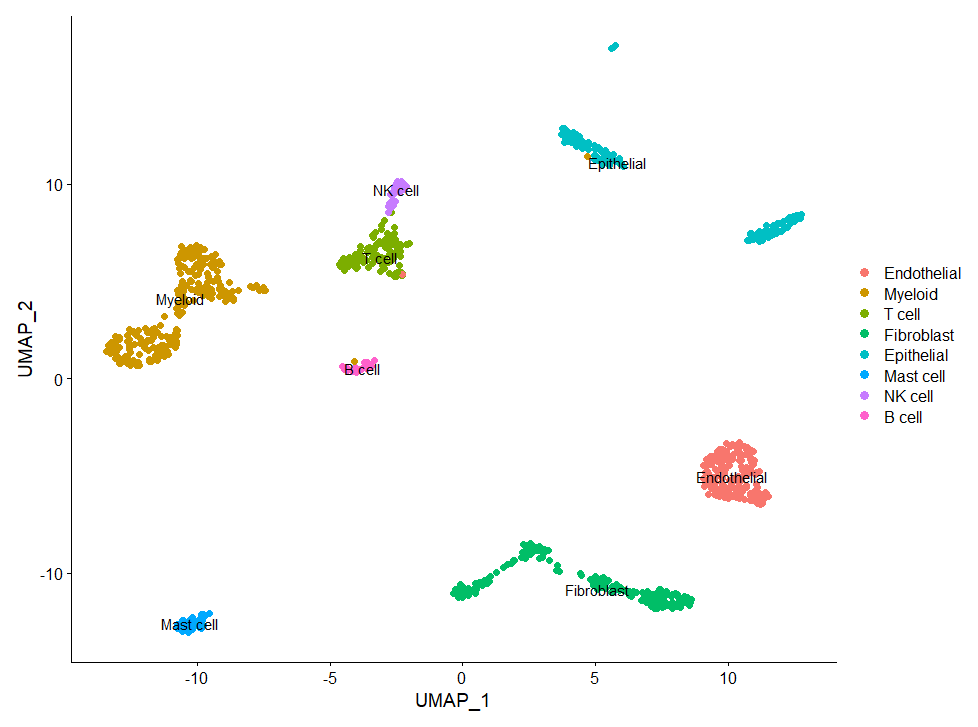

Supplement: Supplementary file 2 [file DataSheet_2.zip › Supplemental Figures/Fig S1/Fig 1B.png]

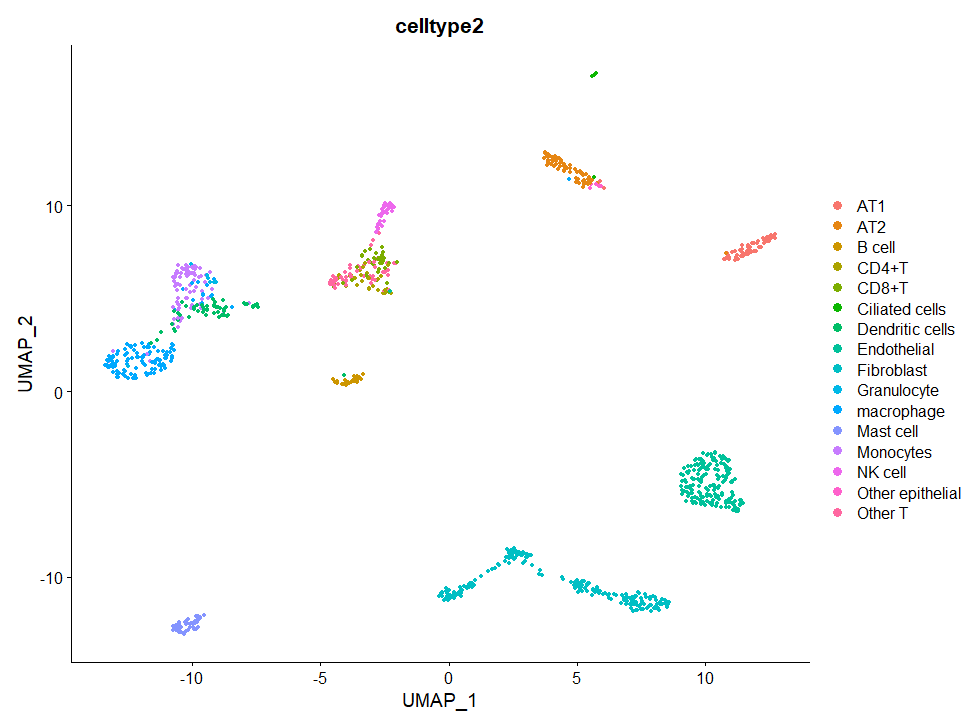

Supplement: Supplementary file 2 [file DataSheet_2.zip › Supplemental Figures/Fig S1/Fig 1A.png]
